# Design and Optimization of Self-Powered Photodetector Using Lead-Free Halide Perovskite Ba_3_SbI_3_: Insights from DFT and SCAPS-1D

**DOI:** 10.3390/nano15211656

**Published:** 2025-10-30

**Authors:** Salah Abdo, Ambali Alade Odebowale, Amer Abdulghani, Khalil As’ham, Yacine Djalab, Nicholas Kanizaj, Andrey E. Miroshnichenko

**Affiliations:** 1 School of Engineering and Technology, University of New South Wales at Canberra, Northcott Drive, Canberra, ACT 2610, Australia; s.abdo@unsw.edu.au (S.A.); a.odebowale@unsw.edu.au (A.A.O.); a.abdulghani@unsw.edu.au (A.A.); k.asham@unsw.edu.au (K.A.); n.kanizaj@unsw.edu.au (N.K.); 2 Physico-Chemistry of Materials and Environment Laboratory, Physics Department, Ziane Achour University of Djelfa, Djelfa BP 3117, Algeria; yacine.djalab@univ-djelfa.dz

**Keywords:** lead-free perovskite, cubic Ba_3_SbI_3_, density functional theory (DFT), photodetectors, SCAPS-1D simulation, responsivity, detectivity

## Abstract

All-inorganic halide perovskites have attracted significant interest in photodetector applications due to their remarkable photoresponse properties. However, the toxicity and instability of lead-based perovskites hinder their commercialization. In this work, we propose cubic Ba_3_SbI_3_ as a promising, environmentally friendly, lead-free material for next-generation photodetector applications. Ba_3_SbI_3_ shows good light absorption, low effective masses, and favorable elemental abundance and cost, making it a promising candidate compound for device applications. Its structural, mechanical, electronic, and optical properties were systematically investigated using density functional theory (DFT) with the Perdew–Burke–Ernzerhof (PBE) and hybrid HSE06 functionals. The material was found to be dynamically and mechanically stable, with a direct bandgap of 0.78 eV (PBE) and 1.602 eV (HSE06). Photodetector performance was then simulated in an Al/FTO/In_2_S_3_/Ba_3_SbI_3_/Sb_2_S_3_/Ni configuration using SCAPS-1D. To optimize device efficiency, the width, dopant level, and bulk concentration for each layer of the gadgets were systematically modified, while the effects of interface defects, operating temperature, and series and shunt resistances were also evaluated. The optimized device achieved an open-circuit voltage (Voc) of 1.047 V, short-circuit current density (Jsc) of 31.65 mA/cm^2^, responsivity of 0.605 A W^−1^, and detectivity of 1.05 × 10^17^ Jones. In contrast, in the absence of the Sb_2_S_3_ layer, the performance was reduced to a Voc of 0.83 V, Jsc of 26.8 mA/cm^2^, responsivity of 0.51 A W^−1^, and detectivity of 1.5 × 10^15^ Jones. These results highlight Ba_3_SbI_3_ as a promising platform for high-performance, cost-effective, and environmentally benign photodetectors.

## 1. Introduction

Photodetectors are light-harvesting photosensors that detect light and instantaneously convert it into an electrical signal [1]. They are available in various forms such as photodiodes, phototransistors, and photoconductors, each tailored to specific applications based on their sensitivity and response speed [2,3,4]. The working principle starts when the device’s active material captures incident photons, generating electron–hole pairs, which are then transported to the device’s contacts, producing a measurable electric current [5]. The ability to translate this light into an electrical signal makes photodetectors vital in modern technology, playing a critical role in sensing, imaging, optical communication, consumer and professional cameras, military systems, and medical imaging [6,7,8,9,10]. The operational range of photodetectors is determined by their design and constituent materials, allowing them to function across various regions of the electromagnetic spectrum, including the ultraviolet (UV), visible, and near-infrared (NIR) domains [11].

Efficient photodetectors are characterized by their high sensitivity, fast response time, and a broad spectral range. This enables them to accurately detect light across various wavelengths. Low noise levels are equally important to ensure clear signal detection, even under low-light conditions [12,13]. Moreover, strong air stability and long-term reliability are essential for durable operation, particularly in highly demanding environments where device performance is critical, such as in medical diagnostics [14]. Additionally, an ideal photodetector operates with low power consumption, enabling seamless integration into compact electronic systems [15]. These performance metrics are largely dictated by the choice of materials. The ideal active material possesses a direct bandgap, a high absorption coefficient, earth-abundant composition, and non-toxic characteristics [16]. However, it is challenging to find a single material that perfectly satisfies all these requirements.

Over the years, a wide range of materials has been investigated for photodetection applications. Silicon (Si) remains the most widely used material due to its affordability, well-established fabrication methods, and excellent response [17]. However, Si possesses an indirect bandgap, which is unfavorable for high-speed photodetection [18]. For detection beyond silicon’s operational range, alternative materials were employed. Indium gallium arsenide (InGaAs) is well-suited for infrared detection, whereas gallium nitride (GaN) and silicon carbide (SiC) are preferred for ultraviolet sensing [10,19,20]. These materials are considered safe and environmentally friendly. Ultimately, the selection of a suitable material, based on its unique optical and electrical properties, remains a fundamental factor in the design and optimization of high-performance photodetectors [21].

Organic–inorganic lead halide perovskites (OILHPs) have gained significant attention for their excellent optoelectronic properties, including broad optical absorption, tunable bandgaps, long carrier diffusion lengths, high mobility, and low-cost synthesis [22,23]. Among these OIHP materials, MAPbI_3_ and MAPbCl_3_ compounds have attracted significant interest in numerous applications such as solar cells, photodetectors, and LEDs [24,25,26]. However, their commercial use is restricted due to their toxicity and poor stability [27]. To address the stability issues, researchers have explored cation engineering, replacing the organic MA^+^ with more stable FA^+^ or inorganic Cs^+^, leading to mixed-cation and all-inorganic perovskites with improved environmental and structural stability [28,29]. Recent works, such as the development of CsPbBr_3_/CdS heterojunction photodetectors exhibiting high responsivity and structural robustness [30] and quasi-2D perovskites employing dual-cation (Cs^+^, Cu^+^) release strategies for defect passivation and ultrafast photoresponse [31], have further highlighted the potential of inorganic cation engineering to enhance both performance and stability. Despite their enhanced stability, lead toxicity remains a concern, driving the search for lead-free perovskites by replacing Pb^2+^ with Sn^2+^ or Ge^2+^. CsSnI_3_, a Sn-based perovskite, has a narrow bandgap suitable for photodetectors, but Sn^2+^ easily oxidizes to Sn^4+^, creating vacancies that generate excess holes. This high hole density gives the material a metallic-like character and weakens its optoelectronic performance [32].

Many research efforts were focused on developing stable, non-toxic alternatives to lead-based perovskites with comparable optoelectronic performance. Bismuth halide perovskites (BHPs) have attracted growing interest due to their environmental safety, high stability, and excellent photoelectric properties, making them suitable for X-ray detectors and neuromorphic computing [33,34]. However, their relatively wide bandgaps of 2.0 eV make them less effective for near-infrared detection. Inorganic chalcogenide perovskites have also emerged as promising semiconductors for photodetection applications because of their excellent thermal and chemical stability. However, these materials are still emerging, and their complex chemistry, along with the need for high-temperature processing, makes uniform thin-film fabrication challenging [27]. The main obstacle lies in producing high-quality thin films from these materials, which demands the development and application of suitable fabrication strategies [35]. Therefore, well-established fabrication methods are required to produce high-quality thin films before being recognized as reliable photodetector materials. On the other hand, perovskite oxides have garnered significant attention due to their exceptional structural stability. However, their relatively wide bandgaps, typically exceeding 2.5 eV, substantially limit their ability to detect visible and near-infrared light. As a result, photodetectors based on these oxides are generally limited to operation within the ultraviolet region [36]. Therefore, developing new lead-free and high-performance materials is essential for advancing next-generation commercial photodetectors.

A new family of inorganic halide perovskites with a formula A_3_MX_3_ family, adopting the Pm$\bar{3}$m (no. 221) space group, where A represents alkaline-earth metals (Sr, Ba, Ca), M denotes pnictogens (Sb, P, As), and X refers to halide anions (I, Cl, Br), has emerged as a promising class of lead-free photovoltaic absorber and photodetector materials [37]. The compounds adopt a unique anion-centered octahedral network configuration ([MX_6_]), very similar to cation-centered octahedra found in conventional ABX_3_ perovskites. This octahedral network configuration contributes to band edge states, which exhibit parity-allowed transitions, enabling efficient light absorption for photovoltaic applications [38]. Their nonstoichiometric composition gives rise to unique structural motifs and bonding environments absent in conventional perovskites [39]. Recent studies have demonstrated that most A_3_MX_3_ compounds possess a direct bandgap, good mechanical stability, and good electronic properties [40]. Although the family had been identified earlier, it attracted limited attention until 2021, when it began to gain significant interest in optoelectronic applications. Experimental studies have already demonstrated the successful synthesis of certain members of this family. The first synthesis of the Mg_3_NF_3_ compound was reported in 1969. This compound was synthesized at high temperatures (1050 °C), exhibiting a high-symmetry cubic structure with a space group of (Pm$\bar{3}$m) [37,41]. Likewise, Ca_3_AsX_3_ (X = Cl, Br, I) compounds have also been experimentally synthesized in the cubic phase at temperatures ranging from 800 to 1000 °C [42,43,44].

Motivated by the feasibility of the synthesis routes for these compounds, Hong-Jian Feng and Qiang Zhang et al. screened 64 compounds from the A_3_MX_3_ family using density functional theory (DFT). Their analysis revealed Ba_3_PI_3_, Ba_3_AsI_3_, and Ba_3_SbI_3_ as higher-performance and stable candidates for photovoltaic applications. Among these three stable compounds, Ba_3_SbI_3_ realized the highest theoretical power conversion efficiency of 25.9% with optical absorption comparable with the champion halide perovskite (MAPbI_3_) and GaAs [37]. The Ba_3_SbI_3_ with lead-free composition exhibits remarkable structural, optical, dynamic, thermal, and electronic properties. This compound is found to be ideally stable under ambient conditions. Its dynamic stability has been confirmed through ab initio molecular dynamics simulations, which show that Ba_3_SbI_3_ maintains its crystal structure under ambient conditions with minimal distortion [37,45]. These attractive features, along with a large optical transition probability, have drawn significant interest in photovoltaic applications. For instance, Rahman et al. used DFT with the GGA-PBE function to predict a bandgap of 0.78 eV, as well as low effective masses with moderate carrier mobility, later modeling a solar device with ZnS_2_ as the electron transport layer, realizing a simulated efficiency of 30.49% using SCAPS-1D [45]. Furthermore, Harun-Or-Rashid et al. predicted a bandgap of 1.0 eV using the GGA-PBE method and modeled a Ba_3_SbI_3_-based solar cell, attaining a PCE of roughly 30.26% [46]. However, the GGA underestimates the bandgap, and since no experimental bandgap measurements are currently available for Ba_3_SbI_3_, several DFT calculations using the HSE06 hybrid functional have been performed to estimate its optical bandgap. Islam et al. reported a bandgap of 1.384 eV using HSE06 and then incorporated this bandgap into a pn-junction simulation, realizing a solar efficiency of 21.29% [47]. Additionally, Liu et al. predicted a direct bandgap of 1.40 eV using HSE06 and reported excellent light absorption coefficients exceeding 10^5^ cm^−1^ across the 2.0–4.0 eV photon energy range [48]. Similarly, Liu et al. used HSE06 and predicted a direct bandgap of 1.512 eV, highlighting Ba_3_SbI_3_’s potential for solar cells and visible-range optoelectronics [49]. In addition, Rahman et al. conducted pressure-dependent DFT simulations, showing that Ba_3_SbI_3_ maintains a direct bandgap of 1.044 eV at 0 GPa using the mGGA functional, which gradually decreases with increasing pressure, leading to a semiconductor-to-metal transition near 10 GPa [50]. Even though the structure of Ba_3_SbI_3_ has not been synthesized yet, it can be realized using appropriate precursors under optimized temperature and annealing conditions, following methods similar to previously reported procedures within the same family [37]. Although Ba_3_SbI_3_ perovskites possess good optoelectronic properties and a suitable bandgap, they have not yet been investigated for photodetector applications. To the best of our knowledge, this study is the first to demonstrate the photodetection properties of Ba_3_SbI_3_, thereby extending the application scope of the A_3_BX_3_ halide perovskite family beyond photovoltaics. These findings highlight the potential of Ba_3_SbI_3_ as a lead-free material for next-generation photodetector applications and open new avenues for exploring the broader A_3_BX_3_ family for various types of photodetectors.

To form a p–n junction with the Ba_3_SbI_3_ compound for photodetector applications, a window layer or ETL layer is needed [51]. This material needs to be transparent, have high boiling and melting points, possess relatively high resistance, and be conductive [52]. Various materials have been reported in the literature as n-type layers for forming *p–n* junctions with different absorbers in photodetector and photovoltaic applications, including In_2_S_3_, SnS_2_, and CdS [52,53,54]. Among these materials, In_2_S_3_ stands out as a window layer with a wide direct bandgap in the range of 2.3–2.7 eV. It possesses several features such as low toxicity, high optical transmittance, high dielectric permittivity, strong chemical stability, and significant light absorption, making it a promising candidate for photodetector applications [55]. Indium sulfide (In_2_S_3_) exhibits intrinsic n-type conductivity and excellent compatibility with a variety of absorber materials, making it a popular and environmentally benign alternative to CdS in thin-film device architectures. Owing to these favorable properties, In_2_S_3_ has been widely employed as a buffer layer to form p–n junctions with diverse absorber materials in thin-film solar cells and photodetectors, where it effectively facilitates charge separation and transport at the heterointerface [52,56]. With an electron affinity of approximately 4.4 eV, indium sulfide (In_2_S_3_) exhibits high optical transparency and favorable energy-level alignment with lead-free perovskite absorbers such as Ba_3_SbI_3_ [52]. The small conduction-band offset (−0.15 eV) between In_2_S_3_ and Ba_3_SbI_3_ enables efficient electron extraction across the heterojunction, while the deep valence-band maximum (−6.75 eV) and the corresponding valence-band offset (+1.12 eV) establish a substantial barrier to hole transport. This energetic configuration could suppress interfacial charge recombination, thereby enhancing carrier selectivity and overall device performance [52]. In_2_S_3_ exhibits moderate electron mobility and can be deposited through low-cost, low-temperature techniques such as photochemical deposition, reactive evaporation, modulated flux deposition, spray pyrolysis, and thermal evaporation [52,57]. These attributes make In_2_S_3_ a viable and practical choice for use as an ETL or buffer layer in Ba_3_SbI_3_-based optoelectronic devices.

It has been demonstrated that incorporating an HTL or back surface field (BSF) layer between the back contact and the absorber layer reduces recombination and enhances the efficiency of the photodetector [51]. In this regard, we propose using antimony sulfide (Sb_2_S_3_) as a BSF layer between the main absorber and the back contact (Ni) to mitigate the minority carrier recombination at the back contact. It is a promising p-type semiconductor possessing a bandgap of approximately 1.7 eV, strong stability, and a high absorption coefficient (~10^5^ cm^−1^), offering several advantages when used as a BSF layer in optoelectronic devices. Sb_2_S_3_, composed of antimony and sulfur, both of which are non-toxic and relatively earth-abundant elements, is well suited for developing environmentally friendly photodetectors [58]. It has been extensively used as a BSF layer to enhance the performance of photodetectors and photovoltaics, making it a subject of interest for developing cost-effective devices [59,60]. With an electron affinity of ~3.8 eV, Sb_2_S_3_ provides a favorable type-II band alignment with Ba_3_SbI_3_, enabling efficient hole extraction and effective electron blocking. Its valence-band maximum (5.5 eV) is closely aligned with that of Ba_3_SbI_3_ (−5.63 eV), resulting in a small valence-band offset (0.134 eV) that enables smooth hole transport with minimal energy loss. Simultaneously, the conduction-band offset (~0.45 eV) suppresses electron back-injection, reducing interfacial recombination at the Ni contact [58,61]. Sb_2_S_3_ thin films can be fabricated using various deposition techniques, including spin coating, atomic layer deposition, thermal evaporation, and chemical bath deposition [62]. Owing to its suitable electronic properties, stability, and compatibility with perovskite absorbers, Sb_2_S_3_ serves as an effective BSF layer for forming heterojunctions with Ba_3_SbI_3_ in photodetector applications.

This study aims to theoretically inspect the feasibility of the lead-free perovskite Ba_3_SbI_3_ compound for photodetector applications. The study starts with examining the structural, electronic, mechanical, and optical properties of Ba_3_SbI_3_ using density functional theory, employing both GGA and HSE06 functionals. Based on these predicted properties, the photoresponse of the Ba_3_SbI_3_-based Al/FTO/In_2_S_3_/Ba_3_SbI_3_/Sb_2_S_3_/Ni device was modeled using SCAPS-1D 3.3.12 simulation software. To achieve optimal photoresponse, several key factors were optimized, including the layer width, carrier level, and defect levels of each layer in the photodetector. In addition, the effects of interface defects, operating temperature, series resistance, and shunt resistance were considered. The impact of these parameters on short-circuit current, open-circuit voltage, quantum efficiency, responsivity, and detectivity was carefully analyzed. This investigation highlights Ba_3_SbI_3_’s potential as a lead-free perovskite for advanced optoelectronic devices beyond photovoltaics, particularly in environmentally benign photodetectors.

## 2. Materials and Methods

### 2.1. DFT Simulation

This study investigates the structural, mechanical, electronic, and optical properties of the Ba_3_SbI_3_ perovskite compound using the CASTEP Academic v25.1.1 (CASTEP Developers’ Group, Cambridge, UK) [63]. The plane-wave cutoff energy and Monkhorst–Pack k-point grid were independently optimized by testing multiple values to minimize the total-energy difference between the initial and final volumes. The parameters producing the smallest deviation were selected, yielding an optimal cutoff energy of 500 eV and a k-point mesh of 8 × 8 × 8, which were subsequently employed for geometry optimization. Ultrasoft pseudopotentials were adopted to improve computational efficiency, and spin polarization was constrained to the non-magnetic state.

Geometry optimization was performed using the Broyden–Fletcher–Goldfarb–Shanno minimization scheme. The optimization converged successfully, with all convergence criteria satisfied. These residual atomic forces were below 0.0025 eV/atom, the maximum stress was less than 0.0025 GPa, atomic displacements were smaller than 1.0 × 10^−4^ Å, and the total-energy change per atom was below 1.0 × 10^−6^ eV [64]. A maximum of 100 self-consistent field cycles was allowed. After the structure was fully relaxed, the electronic properties were examined. Band dispersions were calculated over a 10 eV energy range with fine k-point spacing, and the results obtained with GGA–PBE were refined using the HSE06 hybrid functional. Including HSE06 and norm-conserving pseudopotentials was essential for obtaining a near-realistic estimation of the bandgap and improving the accuracy of the optical spectra. The optical response was derived from the same calculations, highlighting transitions linked to the electronic band edges.

To assess the lattice dynamics, phonon dispersion spectra were calculated using both density functional perturbation theory (DFPT) and the finite-displacement method within supercells, providing a consistent evaluation of the material’s vibrational stability. Mechanical stability was further examined via the stress–strain approach within the generalized gradient approximation (GGA–PBE) framework.

### 2.2. Simulation and Proposed Design

In this section, the simulation approach and proposed design of the Ba_3_SbI_3_-based photodetector are presented. Numerical simulations are important tools that can be harnessed to optimize the device structure before experimental validation. Several advanced tools have been used in the literature to evaluate and enhance photodetector performance, such as TCAD, ANSYS Lumerical Charge, COMSOL Multiphysics, and SCAPS-1D [5,51,65,66]. SCAPS-1D, developed by Marc Burgelman at Ghent University, stands out from other software packages because of its open-source accessibility and user-friendly interface [67]. It has been extensively applied in photovoltaic research, consistently demonstrating good agreement with experimental parameters [68]. Furthermore, SCAPS-1D has proven reliable applications in modeling various photodetector structures such as p–n junction, Schottky, and n–i–p configurations, confirming its suitability for photodetector performance analysis [51,69,70]. Notably, SCAPS-1D enables the simulation of photodetectors with up to seven layers, facilitating the exploration of multilayer architectures and heterojunction designs [71]. It enables the investigation of material and device parameters such as layer width, doping concentrations, defect densities, work functions, and bandgap energies [51].

In this study, SCAPS-1D software was used to analyze the photoresponse characteristics of the Ba_3_SbI_3_-based device and compute the photodetector key outputs, including current–voltage (J–V) characteristics, quantum efficiency (QE), and doping volume, thereby providing valuable insights into carrier dynamics and device optimization [54]. SCAPS-1D solves the fundamental semiconductor equations, including electrons and holes, Poisson’s equation, and the continuity equations, to compute voltage, photocurrent, and QE, offering a reliable photodetector simulation framework [72].

(1) $$\frac{d^{2}\Psi }{dx^{2}}=\frac{q}{\varepsilon_{s}}\left[p\left(x\right)-n\left(x\right)+N_{d}^{+}-N_{a}^{-}+p_{t}\left(x\right)-n_{t}\left(x\right)\right]$$

(2) $$\frac{\partial_{n}\left(x,t\right)}{\partial_{t}}=\frac{I}{q}\frac{\partial J_{n}}{\partial x}+G_{n}\left(x\right)-R_{n}\left(x\right)$$

(3) $$\frac{\partial_{p}\left(x,t\right)}{\partial t}=\frac{I}{q}\frac{\partial J_{p}}{\partial x}+G_{p}\left(x\right)-R_{p}\left(x\right)$$

(4) $$J_{n}=q\mu_{n}n\frac{\partial_{\emptyset }}{\partial x}+qD_{n}\frac{\partial_{n}}{\partial x}$$

(5) $$J_{P}=q\mu_{p}p\frac{\partial_{\emptyset }}{\partial x}-qD_{P}\frac{\partial_{P}}{\partial x}$$

where Ψ stands for the electrostatic potential, and q denotes the electron charge.

The permittivity of the material is represented by $\varepsilon_{s}$. $N_{d}^{+}$ and $N_{a}^{-}$ signify the concentrations of donor and acceptor, respectively. The concentrations of holes and electrons are denoted by $p\left(x\right)$ and $n\left(x\right)$, respectively. $p_{t}\left(x\right)$ represents the spatial distribution of the holes, while $n_{t}\left(x\right)$ defines the spatial distribution of electrons. $J_{n}$ and $J_{p}$ represent the electron and hole current densities, respectively. $G_{p}\left(x\right)$ indicates the total carrier generation rate, while $R_{p}\left(x\right)$ determines the recombination rate. $D_{n}$ signifies the electron diffusion coefficient, while $D_{P}$ represents the hole diffusion coefficient. The current densities of electrons and holes are indicated by $J_{n}$ and $J_{P}$, respectively.$\;\mu_{n}$ corresponds to electron mobility, while $\mu_{p}$ signifies hole mobility [73].

**Figure 1:**
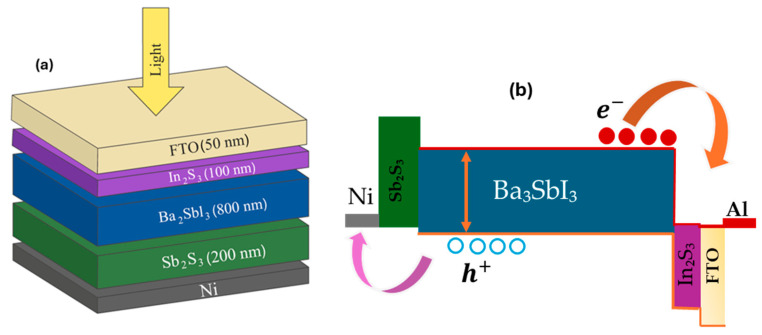
(**a**) Structure of proposed Al/In_2_S_3_/Ba_3_SbI_3_/Sb_2_S_3_/Ni photodetector; (**b**) energy band alignment diagram of the photodetector device incorporating FTO, In_2_S_3_, Ba_3_SbI_3_, and Sb_2_S_3_ layers.

Figure 1a presents the schematic architecture of the designed photodetector, which employs the FTO/In_2_S_3_/Ba_3_SbI_3_/Sb_2_S_3_/Ni multilayer configuration. Each layer in this structure fulfills a distinct functional role, contributing to optimized charge transport and enhanced interfacial stability. In this structure, fluorine-doped tin oxide (FTO) functions as the transparent conductive oxide (TCO), providing a transparent front contact. In_2_S_3_ is employed as the electron transport layer (ETL)/window layer, facilitating efficient extraction of photogenerated electrons. The central light-absorbing layer is Ba_3_SbI_3_, chosen for its novelty and favorable optoelectronic properties. It also offers additional advantages, such as being non-toxic, low-cost, and earth-abundant [37]. Sb_2_S_3_ is used as the BSF to selectively transport holes toward back contact. Nickel (Ni), with a work function of 5.35 eV, was used as the back electrode to complete the device structure and enhance charge extraction in conjunction with the FTO front contact (4.2 eV). The selection of In_2_S_3_ as the window layer and Sb_2_S_3_ as the BSF layer ensures favorable energy-level alignment, facilitates efficient carrier transport, and enhances the potential for integration into flexible optoelectronic devices [54,74].

The band alignment at the interfaces between adjacent layers of the photodetector is a key factor influencing carrier transport, charge separation, and overall device efficiency. Proper alignment of conduction and valence bands enables smooth carrier injection and extraction, minimizing recombination and enhancing the device efficiency. Conversely, unfavorable offsets create barriers that hinder charge flow and degrade photodetector performance. Thus, careful band engineering across multilayer heterojunctions is essential for achieving high performance and operational stability [51]. The band alignment diagram of the proposed FTO/In_2_S_3_/Ba_3_SbI_3_/Sb_2_S_3_/Ni device is illustrated in Figure 1b. The structure exhibits a type-II band alignment that enables efficient carrier separation and transport [75]. The In_2_S_3_/Ba_3_SbI_3_ interface shows a cliff-like alignment that facilitates electron transfer toward the FTO and Ag contact while suppressing backflow. Similarly, the Ba_3_SbI_3_/Sb_2_S_3_ junction presents moderate conduction- and minimal valence-band offsets, favoring hole extraction toward the Ni back contact while blocking electron flow. The Sb_2_S_3_/Ni interface features nearly aligned energy levels, ensuring effective hole collection. Such a staggered band alignment facilitates charge carrier separation and transfer, ultimately improving photodetector efficiency. Under the illumination, excitons generated in the absorber are effectively directed toward the ETL and HTL and subsequently collected by the metal contacts. The CBO, VBO, EC, and EV for each layer of the heterostructures are further detailed in Section 1 of the Supplementary Materials.

The optoelectronic performance of the designed photodetector was first simulated at 300 K, under standard illumination with light intensity of 100 mW/cm^2^ and a frequency of 1.0 × 10^6^ Hz. The temperature was then systematically varied to evaluate its impact on key device metrics. The illumination area was fixed at 1.0 cm^2^, consistent with the standard configuration employed in SCAPS-1D simulations using AM 1.5G solar illumination (100 mW·cm^−2^) [52]. The voltage in the SCAPS-1D software was swept from 0 to 1.3 V in 0.02 V steps to analyze the current–voltage (J–V) characteristics. The QE was determined using SCAPS-1D, enabling detailed figures of merit for extraction devices such as Voc and Jsc. Wavelength-dependent simulations were also conducted to investigate the spectral QE, responsivity, and detectivity under different thermal conditions. The physical and optoelectronic properties of the materials used in the photodetector structure are summarized in Table S1.

The responsivity (A/W), a key performance indicator for photodetectors, is determined using Equation (6), enabling the assessment of how efficiently the device converts incident light into electrical current [76]:

(6) $$R=\frac{\;\eta *\lambda \;\left(nm\right)}{1240\;\left(nm\right)}=\frac{\;J_{ph}}{P_{o}}$$

Here, η signifies the quantum efficiency, λ corresponds to the wavelength of incident light, $J_{\mathrm{ph}}$ denotes the photo-induced current density (A·cm^−2^), and P_0_ represents the optical power incident on the device surface (mW·cm^−2^).

The specific detectivity (Jones) is another critical metric used to assess a photodetector’s performance. It is obtained using Equation (7) [77]:

(7) $$D^{*}=\frac{R}{\sqrt{2qJ_{o}}}$$

The symbol q denotes the elementary charge, while J_0_ refers to the reverse saturation current density. To assess the operation of the photodetector under zero-bias conditions, J_0_ is determined according to Equation (8) [76]:

(8) $$J_{o}=\frac{J_{sc}}{e{(}^{V_{oc}}/0.026)-1\;}$$

The values of Jsc and Voc were obtained from the SCAPS-1D simulation software [52].

## 3. Results

### 3.1. DFT Calculations

#### 3.1.1. The Structural Properties of the Ba_3_SbI_3_ Compound

**Figure 2:**
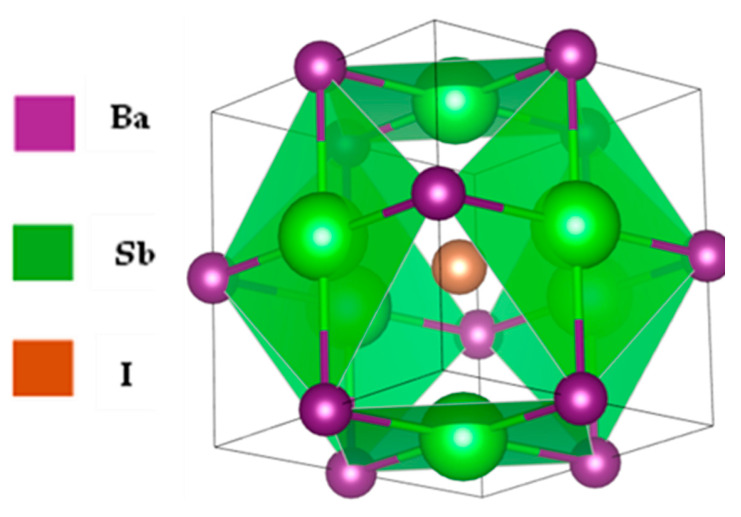
Crystal structure of relaxed Ba_3_SbI_3_ compound in 3D visualization.

Figure 2 portrays the structural characteristics of Ba_3_SbI_3_, a perovskite compound that crystallizes in the cubic phase, adopting the Pm$\bar{3}$m (No. 221) space group [37,78]. The structure consists of unit cells, each involving nineteen atoms, where Ba atoms occupy the face-centered positions at fractional coordinates (0.5, 0.5, 0), while the Sb atom is located at the body-centered Wyckoff position (0.5, 0.5, 0.5). Iodine atoms are situated at Wyckoff positions (0.5, 0, 0), corresponding to the centers of the unit cell edges [79]. The lattice parameter of the optimized Ba_3_SbI_3_ structure is 5.05 Å. This value shows good agreement with previous studies under ambient conditions, validating the accuracy of our DFT calculations. The structural parameters from this study, as well as those reported in earlier works, are summarized in Table 1.

As a cubic perovskite (ABX_3_) material, Ba_3_SbI_3_ exhibits the conventional perovskite framework, where Ba^2+^ cations occupy the cube corners, Sb^3+^ cations reside at the body center, and I^−^ anions are positioned at the face centers. This arrangement produces a three-dimensional network of corner-sharing [SbI_6_]^3−^ octahedra, characteristic of perovskite lattices. Each Sb atom is octahedrally coordinated by six I^−^ ions, forming a highly symmetric and stable lattice with minimal distortion. The uniform Ba–I coordination further reinforces the ideal cubic symmetry, while the periodic alignment of [SbI_6_]^3−^ octahedra underpins the fundamental geometric framework typical of perovskite materials.

The structural stability of the Ba_3_SbI_3_ perovskite can be evaluated using the Goldschmidt tolerance factor (t), a common criterion for assessing perovskite geometries. The tolerance factor is defined by Equation (9) [47]:

(9) $$t=\frac{r_{A}+r_{x}}{\sqrt{2}(r_{M}+r_{X})}$$

where the halide anion (I), B-site cation (Sb), and A-site cation (Ba) are denoted as $r_{X}$, $r_{M}$, and $r_{A}\;$, respectively. Perovskite structures are most likely to form when (*t*) lies within the range of 0.8–1.0 [46]. In previous work [46], the ionic radii of perovskite structure Ba_3_SbI_3_ were reported as $r_{A}$ = 1.42, $r_{M}$ = 0.71 Å, and $r_{X}\;$ = 2.20 Å. Using these values, the tolerance factor was calculated as t = 0.865, which falls within the ideal stability range, confirming the possibility of forming a perovskite phase. Table 2 shows the summary of the calculated tolerance factor of the Ba_3_SbI_3_ compound.

#### 3.1.2. The Elastic and Mechanical Properties of the Ba_3_Sbl_3_ Compound

The elastic properties of the perovskite Ba_3_SbI_3_ are examined in this section. The elastic constants of a compound play a central role in determining its mechanical behavior. These constants are typically obtained through first-principles simulations by applying small deformations to the relaxed crystal structure and calculating the resulting internal stresses [64]. Understanding the elastic constants (Cij) provides crucial details about how a material reacts to an external strain. For cubic crystals, there are three independent components of the elastic constant tensor: (C11, C12, C44). The three independent elastic constants for cubic perovskite (C11, C12, and C44) were obtained and are listed in Table 3. Key elastic properties can be obtained using these constants, but first, we must use the Born stability criterion to assess the material’s stability [80]: $C_{11}>0,C_{44}>0,C_{11}-C_{12}>0,C_{11}+2C_{12}>0,{\;\mathrm{and}\;C}_{12}<B<C_{11}$.

After using the equations above, we concluded that the cubic perovskite under investigation meets the stability requirements. As previously mentioned, several mechanical characteristics, including the bulk modulus (B), shear modulus (G), Young’s modulus (E), anisotropic factor (A), Poisson’s ratio (v), and Pugh’s index ratio (B/G), can be obtained using the elastic constants. We use the Hill average for those parameters whose equations are as follows [81]:

The bulk modulus is calculated using Equation (10)

(10) $$B=\frac{C_{11}+2C_{12}}{3}$$

The Shear modulus (Hill average) is determined using Equation (11)

(11) $$G=\frac{G_{v}+G_{R}}{2}$$

The Voigt shear modulus $G_{V}$ is given by the following Equation (12):

(12) $$G_{V}=\frac{C_{11}-C_{12}+3C_{44}}{5}$$

The Reuss shear modulus is calculated using Equation (13):

(13) $$G_{R}=\frac{5\left(C_{11}-C_{12}\right)C_{44}}{4C_{44}+3\left(C_{11}-C_{12}\right)}$$

The Young’s modulus is obtained using Equation (14):

(14) $$E=\frac{9GB}{3B+G}$$

The anisotropic factor (A) is given by Equation (15):

(15) $$A=\frac{2C_{44}}{C_{11}-C_{12}}$$

Poisson’s ratio (v) is calculated using Equation (16):

(16) $$\nu =\frac{3B-2G}{2\left(2B+G\right)}$$

Table 3 displays the computed values for (B), (G), (E), (A), (Au), (ν), and (B/G). Positive bulk modulus values, a measure of material hardness, can be used to confirm the intrinsic hardness of Ba_3_SbI_3_. Ba_3_SbI_3_ is a rather soft material with a low resistance to volume change under hydrostatic pressure, as indicated by its bulk modulus of 22.17 GPa. Young’s modulus (E) and shear modulus (G) can be used to further assess a material’s hardness. Young’s modulus shows the stiffness of the material, while the shear modulus shows the shear resistance to externally applied force [82]. For Ba_3_SbI_3_, the values of G = 13.07 GPa and E = 32.77 GPa indicate moderate stiffness.

The anisotropy factor (A) is the main criterion for successfully distinguishing between isotropic and anisotropic properties of materials. A compound is said to be isotropic if A = 1 and anisotropic if A is more than or less than 1 [83]. Here, A = 0.372, which indicates that Ba_3_SbI_3_ is anisotropic, as shown in Table 3. Additionally, substantial elastic anisotropy is shown by the universal anisotropy index (AU = 1.284), suggesting that the mechanical properties are slightly direction-dependent.

In addition, we may estimate the sorts of bonding in a material using the Poisson ratio. Covalent bonding is specified by small Poisson’s ratios; for metallic bonds, this is roughly 0.33, whereas for ionic materials, the average value for (v) is 0.25 [84]. Table 3 shows that the Poisson ratio for Ba_3_SbI_3_ is about 0.25, which suggests a strong ionic connection. The (G/B) ratio is another way we can verify our findings. Ionic materials have a value of 0.6, covalent materials have a value of 1.1, and metallic materials have a value of 0.4. The (G/B) ratio in our case is 0.6, confirming the ionic nature of the material being studied.

Furthermore, the ratio (B/G) proposed by Pugh [85] can be used to study the brittleness and ductility of our materials. Under external stress, brittle materials experience minimal plastic deformation, while ductile materials experience significant plastic deformation. Materials are classified as brittle if their (B/G) ratio is less than 1.75 and as ductile if it is greater than 1.75. We observe that Ba_3_SbI_3_ has a Pugh value of 1.70 according to our computation, indicating that our material is rather brittle.

Our calculated elastic constants and mechanical parameters of the cubic Ba_3_SbI_3_ compound agree excellently with previous theoretical reports, with deviations of less than ~5%, confirming the reliability of our results.

#### 3.1.3. Electronic, DOS, and Phonon Dispersion Characteristics of Ba_3_Sbl_3_ Compound

This section probes the electronic band structure, DOS, and phonon dispersions of Ba_3_SbI_3_ using first-principles calculations to assess its feasibility for photodetector applications. The electronic band structure of a semiconductor critically influences its fundamental physical properties, including bandgap, charge transport, and optical absorption [86]. The size and nature of the bandgap, whether direct or indirect, can also be governed by its band structure. Semiconductor materials with a direct bandgap are generally preferred over those with an indirect bandgap for certain optoelectronic applications, such as solar cells, LEDs, and photodetectors, as they enable strong photon absorption and emission without requiring phonon involvement [87].

**Figure 3:**
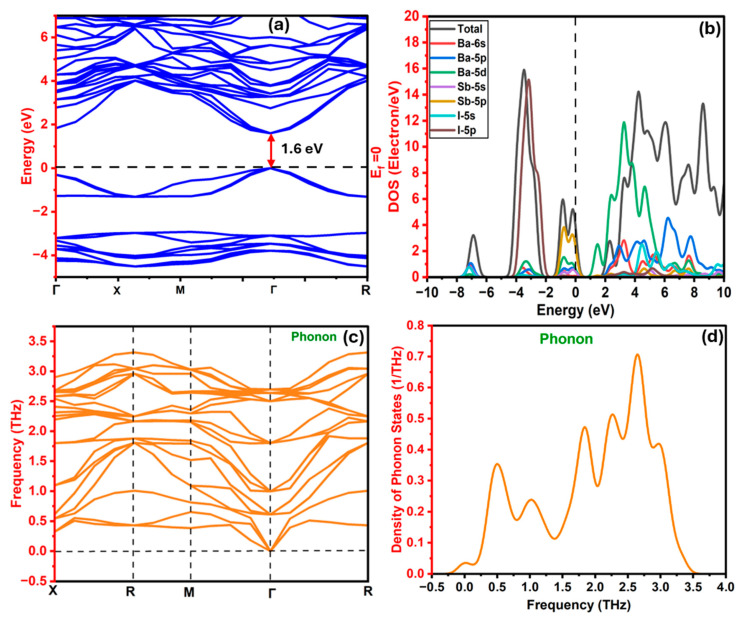
(**a**) Electronic band structure of Ba_3_SbI_3_ compound; (**b**) partial density of states (PDOS) for I, Sb, and Ba atoms, along with the total density of states (TDOS); (**c**) phonon dispersion curves plotted as a function of the high-symmetry directions in the Brillouin zone; (**d**) phonon density of states (PDOS) as a function of frequency.

The band structure of the Ba_3_SbI_3_ compound was first relaxed and optimized using the GGA-PBE functional. After relaxation, a direct bandgap of approximately 0.78 eV was obtained at the Γ point. This value is in strong agreement with previous reports on the same compound [45]. However, it is well established that although GGA-PBE provides reliable accuracy for estimating material properties such as optical and mechanical characteristics, it tends to underestimate the bandgap [87]. The HSE06 functional often provides bandgap predictions that closely match experimental values. To obtain a more precise value, the HSE06 hybrid functional was applied. The HSE06 functional is computationally expensive, and performing full relaxation with it is time-consuming. Therefore, HSE06 was applied using the relaxed structure obtained from GGA-PBE calculations. Using HSE06, a direct bandgap of 1.60 eV is realized at the Γ point, as shown in Figure 3a. The calculated band structure was traced along the high-symmetry path X–R–M–Γ–R in the Brillouin zone, with the Fermi level set at 0 eV and the energy range defined from −4.5 eV to 6.5 eV. It is worth mentioning that the spin–orbit coupling (SOC) effect was not included in our HSE calculations due to limitations in the CASTEP code. Although HSE06 may still slightly underestimate the bandgap, its balance of accuracy and computational efficiency confirms Ba_3_SbI_3_ as a promising direct-bandgap material for photodetectors.

Figure 3b provides a detailed analysis of the total and partial density of states (DOS) for the Ba_3_SbI_3_ compound. It offers more insights into the electronic structure, especially the contributions of individual atomic orbitals to the valence and conduction bands [88]. Analyzing the partial DOS helps identify which atoms and orbitals are predominant near the Fermi level, clarifying the roles of Ba, Sb, and I in shaping the compound’s electronic and optical properties. As shown in Figure 3b, the total density of states (TDOS) approaches zero at the Fermi level (E_f_ = 0 eV), confirming the semiconducting nature of Ba_3_SbI_3_. The valence band (−8 eV to 0 eV) is dominated by strong I-p peaks (~15 states/eV), with additional contributions from Sb-p and Sb-s states, reflecting significant bonding interactions. In the conduction band (0 to ~8 eV), Ba-d orbitals dominate, peaking at ~11.9 states/eV, alongside Sb-p and I-s states, indicating orbital hybridization above the Fermi level. Minor contributions from Ba-s and Ba-p states are present in both regions. These features provide further information about the directional bonding and semiconducting behavior of Ba_3_SbI_3_. The bandgap values of Ba_3_SbI_3_, as summarized in Table 4, align with previous studies while considering different cutoff energies and k-point grids.

Besides the electronic properties, the dynamic stability of the material was also studied through phonon dispersion calculations. Phonon characteristics are key to understanding crystalline solids. Information on phase transitions, structural stability, and how lattice vibrations affect charge transport and heat conduction can be effectively extracted from phonon dispersion spectra (PDS) [78,89,90]. Phonon dispersion shows the relationship between a phonon’s energy and its momentum within the crystal lattice [79]. Figure 3c displays the calculated phonon dispersion curve of Ba_3_SbI_3_ using the GGA functional. It is plotted over the frequency range of −0.5 to 3.75 THz along the high-symmetry path X–R–M–Γ–R. The lack of imaginary frequencies in this spectrum confirms the dynamic stability of this compound [49]. In the dispersion curves, the acoustic branches start from the Γ point at zero frequency and increase gradually, while the optical branches reach up to about 3.4 THz. Flat regions seen in the optical modes suggest low phonon group velocities, which may restrict heat transfer. Furthermore, the narrow gap between the acoustic and optical branches indicates potential interactions between these modes, which could affect phonon scattering and thermal conductivity [91].

Figure 3d presents the phonon density of states (PDOS) for Ba_3_SbI_3_ over the range −0.25 to 3.5 THz. Most phonon states are concentrated between 0.25 and 3.0 THz, with the most prominent peak appearing near 2.8 THz. Peaks in the PDOS are associated with specific vibrational patterns in the lattice, where the low-frequency range mainly corresponds to acoustic modes, and the higher-frequency peaks are linked to optical phonons. This pattern suggests that low-frequency acoustic vibrations are the primary heat carriers, whereas optical modes contribute significantly to phonon scattering, thereby affecting the material’s thermal properties [91].

#### 3.1.4. The Optical Properties of the Ba_3_SbI_3_ Compound

The optical properties of the semiconductor offer essential insights into the material’s potential for optoelectronic applications such as photodetection and photocells. They arise from the interaction of light with the material [16], leading to several measurable parameters such as reflectivity, absorption coefficient, refractive index, dielectric function, optical conductivity, and energy loss function. In this study, we calculated and analyzed the optical spectra for photon energies up to 8 eV for each case, except for the energy loss function, which is plotted from 0 to 35 eV to display its full spectral range. All optical properties of the compound were computed using a plane-wave method with the PBE-GGA functional.

**Figure 4:**
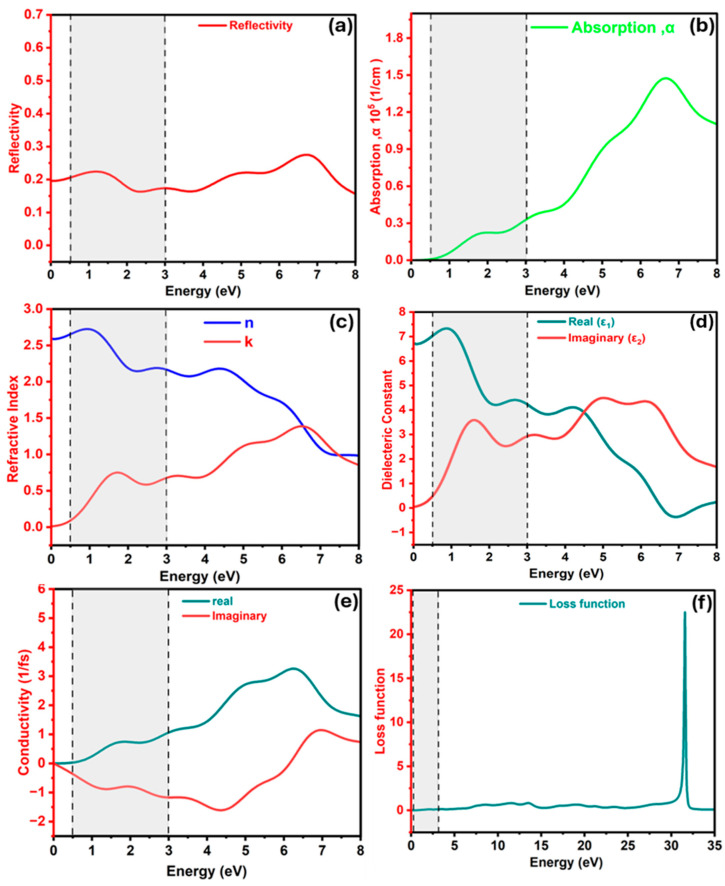
Optical properties of the compound: (**a**) absorption coefficient; (**b**) reflectivity; (**c**) refractive index (n) (blue curve) and extinction coefficient (k) (red curve); (**d**) real component (cyan curve) and imaginary component (red curve) of the dielectric constant; (**e**) energy-dependent real part (red curve) and imaginary part (cyan curve) of the optical conductivity; and (**f**) loss function with respect to energy.

Figure 4a presents the energy-dependent reflectivity spectrum of Ba_3_SbI_3_. The reflectivity remains low throughout the entire spectral region, exhibiting two small peaks of roughly 0.22 and 0.275 at 1.20 eV and 6.7 eV, respectively [4]. Within this range, the reflection spectrum shows slight oscillations but generally maintains low values. The consistently low reflectivity in the visible region signifies high optical transparency and strong photon absorption capability. Therefore, Ba_3_SbI_3_ could absorb a significant fraction of incident photons, which is advantageous in photodetector applications [92].

In optoelectronic research, the absorption coefficient α(ω) is a key parameter for assessing a material’s ability to harvest photons, as it directly determines the depth of light penetration [93]. It also demonstrates the potential of the compound to enable efficient photoexcitation, which is a crucial parameter for identifying high-performance photodetector materials [50]. The optical α(ω) of the Ba_3_SbI_3_ perovskite compound as a function of photon energy is shown in Figure 4b. The spectrum exhibits a continuous rise starting from near-zero photon energy. In the infrared region below 1 eV, the absorption coefficient α(ω) remains below 10^4^ cm^−1^ and then increases, reaching roughly 0.33 × 10^5^ cm^−1^ around 3 eV. A further increase is realized toward higher energies within the measured range, reaching a maximum value of 1.47 × 10^5^ cm^−1^ at 7 eV. Beyond this value, it decreases gradually to 1.1 × 10^5^ cm^−1^ at 8 eV. Generally, the broad spectral coverage from the higher-energy region to the near-UV region underscores the intense light–matter coupling in the Ba_3_SbI_3_ material and highlights its promise for broadband optoelectronics.

Figure 4c exhibits the refractive index n(ω) and the extinction coefficient k(ω) of Ba_3_SbI_3_, which are fundamental parameters describing light–matter interactions. The real part, n, expresses the phase velocity of light and governs optical transparency, whereas the imaginary component, k, quantifies light absorption within the material [49,94]. At zero energy, the static refractive index is relatively high, about 2.60, and increases to a maximum value of 2.72 at 1.2 eV. Beyond this point, it gradually decreases toward unity at higher photon energies. The extinction coefficient starts near zero and remains very small in the 0–2.5 eV range. At higher photon energies, however, k(ω) rises sharply and reaches a peak of 1.39 at 6.6 eV. The combined behavior of n and k therefore highlights that Ba_3_SbI_3_ possesses favorable optical properties, emphasizing its potential for photodetector applications.

Figure 4d depicts the real ε_1_(ω) and imaginary ε_2_(ω) parts of the dielectric function for the Ba_3_SbI_3_ compound. It is often called dielectric permittivity, and it quantifies a material’s ability to hold electrical energy through polarization [95]. At zero energy (eV), the real dielectric constant ε_1_(ω) is around 6.74, which is high enough to be used as a photoabsorber layer for photodetector applications. It also increases and reaches its uppermost value of about 7.33 at 0.87 eV. Beyond this point, it drops with small oscillations and then crosses zero energy at 6.54 eV. In general, the real dielectric function exhibits larger values at lower photon energies compared to its values at higher photon energies. The imaginary dielectric function, ε_2_(ω), provides insight into the electronic bandgap [96]. It exhibits two peaks, a sharp peak of 3.5 at 1.5 eV and a broad maximum peak of about 4.49 at 5.03 eV. At higher energies within the spectral range, ε_2_(ω) drops off, signifying increased absorption losses, as observed previously in Figure 4b of the optical absorption. This high static dielectric constant signifies very low energy losses, making Ba_3_SbI_3_ a promising candidate for photodetector applications [50].

The real (σ_1_) and imaginary (σ_2_) components of the optical conductivity for Ba_3_SbI_3_ are shown in Figure 4e. Optical conductivity is a key parameter for illustrating the electromagnetic response of solids. This parameter plays a vital role, since it governs both the number of photons capable of propagating through the medium and the strength of their interaction with matter [95]. The real part starts at zero and then rises rapidly, reaching its maximum peak of 3.25 (1/fs) at 6.28 eV. The near-zero onset of the real component indicates the semiconducting nature of the Ba_3_SbI_3_ compound [64]. The imaginary part starts with a negative value and shows a minimum value of −1.61 (1/fs) at 4.1 eV. With a further increase in photon energy, σ_2_ recovers, crosses zero again at 6.1 eV, and then attains the highest positive maximum peak of +1.61 (1/fs) at 6.95 eV.

Figure 4f illustrates the loss function *L(ω)* of the Ba_3_SbI_3_ compound with respect to photon energy. It is an essential factor in evaluating the optical properties of the substance. This function describes the energy dissipated by fast electrons as they traverse the medium. In the low-energy range, *L(ω)* remains close to zero, signifying the absence of significant scattering processes below the bandgap as the light interacts with the material [47]. As the photon energy increases, the loss function shows a slight fluctuation in a range from 0.61 to 0.83 across the 7.5–20 eV region. The uppermost peak of 22.51 is observed at 31.57 eV, which occurs when the bandgap of the material becomes less than the photon energy [46]. The low-loss function in the ultraviolet and infrared regions could effectively minimize parasitic optical losses, thereby supporting efficient performance in photodetector applications [79].

### 3.2. Photoresponse of the Ba_3_Sbl_3_ Compound

#### 3.2.1. The Absorption Coefficient for All Layers of the Device

**Figure 5:**
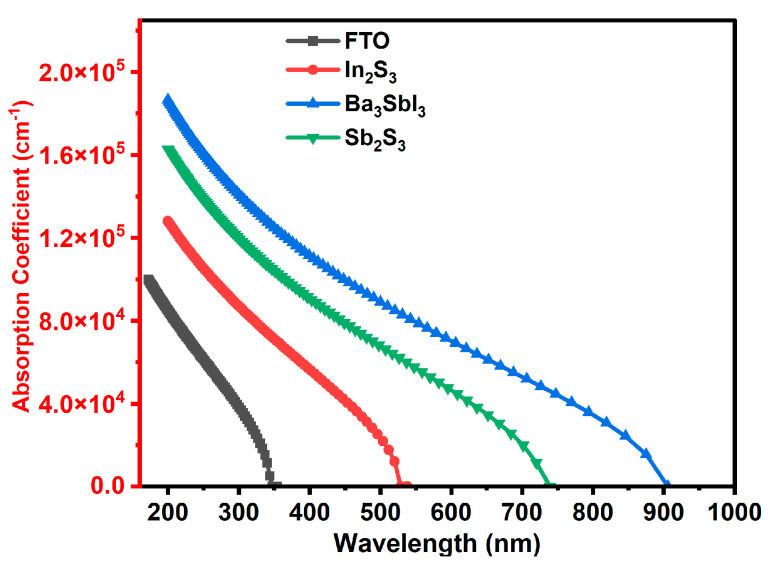
Absorption coefficient of Al/FTO/SnS2/Ba_3_SbI_3_/MoS_2_/Au photodetector structures with FTO, In_2_S_3_, Ba_3_SbI_3_, and Sb_3_S_3_ materials.

The optical absorption coefficient is a crucial parameter in determining how efficiently a material interacts with incident light, and it plays a key role in optimizing photodetector performance. The initial absorption peak is significant, as it identifies the specific wavelength region where maximum light absorption occurs [51]. Figure 5 presents the absorption coefficient spectra α(λ) of FTO, In_2_S_3_, Ba_3_SbI_3_, and Sb_2_S_3_, obtained using SCAPS-1D software. These spectra highlight distinct optical characteristics that determine the suitability of each material for photodetector applications. The FTO material with a bandgap of 3.6 eV exhibits the lowest absorption, with a sharp cutoff near 350 nm, confirming its role as a transparent conductive oxide. In_2_S_3_ reveals moderate absorption extending up to 530 nm, which corresponds to the relatively wide bandgap of 2.35 eV, making it a suitable buffer layer. Sb_2_S_3_ with a bandgap of 1.7 eV, used as the BSF/HTL layer, displays a strong absorption coefficient exceeding 1.6 × 10^5^ cm^−1^ in the UV region. It also maintains strong absorption throughout most of the visible range, with its absorption edge at 745 nm.

Ba_3_SbI_3_, with a bandgap of 1.384 eV, acts as the primary absorber and shows the highest absorption across most of the measured spectrum, exceeding 1.6 × 10^5^ cm^−1^ in the UV range. It also maintains strong absorption deep into the visible and near-infrared spectrum up to 900 nm. This allows for efficient capture of a wide portion of the visible spectrum and promotes significant photocurrent generation. Consequently, the multilayer arrangement of these compounds can ensure effective light harvesting, better carrier collection, and enhanced photodetector performance.

#### 3.2.2. The Ba_3_Sbl_3_-Based PD with and Without the Sb_2_S_3_ Layer

**Figure 6:**
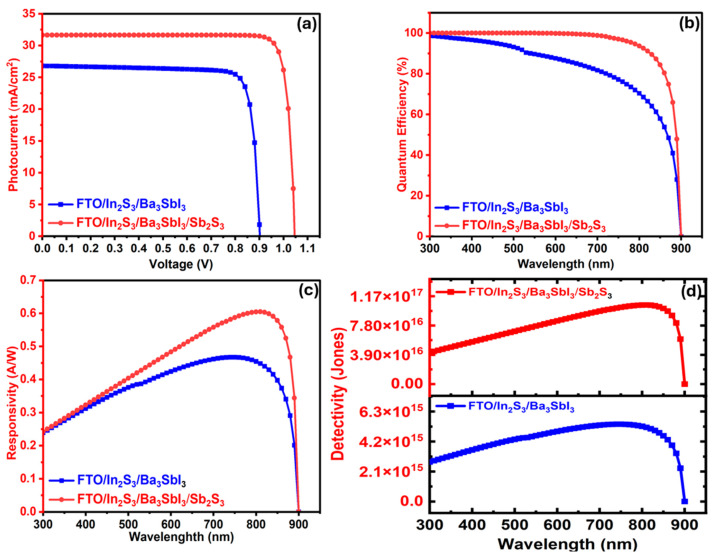
Influence of the Sb_2_S_3_ thin film on the operational characteristics of the detector. Performance comparison for structures without (blue curve) and with (red curve) the Sb_2_S_3_ film: (**a**) J–V characteristics; (**b**) QE; (**c**) photoresponsivity; and (**d**) detectivity.

This section investigates how adding the BSF layer Sb_2_S_3_ to the FTO/In_2_S_3_/Ba_3_SbI_9_ affects the photodetector characteristics. As the BSF layer is added to the structure to form FTO/In_2_S_3_/Ba_3_SbI_9_/Sb_2_S_3_, the performance is significantly enhanced. As shown in Figure 6a, the Jsc grows from 26.8075 mA/cm^2^ to 31.6554 mA/cm^2^, while the Voc is optimized from 0.9017 V to 1.047 V. The improvement in Jsc with the insertion of the BSF layer is due to the formation of a P/P^+^ heterojunction between the Ba_3_SbI_9_ and Sb_2_S_3_ layers, which leads to a reduction in recombination losses at the Ba_3_SbI_9_/Sb_2_S_3_ interface [51]. This results in better carrier transfer, which arises from the enhanced bandgap alignment [97]. On the other hand, the improvement in Voc can be explained by Equation (17), which relates Voc to the ratio of photogenerated current Jsc and dark saturation current (J_0_) [97].

(17) $$V_{oc}=\frac{\;K_{B}*T}{q}ln\frac{\;J_{sc}}{J_{o}}$$

where increasing Jsc or reducing J_0_, as achieved with Sb_2_S_3_, directly enhances Voc.

Without the Sb_2_S_3_ layer, the photodetector exhibits over 97% QE in the 300–500 nm range, which gradually decreases beyond this region, falling to approximately 28% at 890 nm. However, installing the Sb_2_S_3_ layer significantly improves spectral response, achieving 100% QE between 300 and 500 nm, maintaining this level up to 590 nm, and maintaining around 99% from 600 to 890 nm. Sb_2_S_3_ forms a favorable band alignment with the Ba_3_SbI_9_ absorber, enabling selective blocking of majority carriers and redirecting them toward the front contact. This carrier-selective behavior reduces recombination losses and results in improved QE and overall photodetector performance [54].

Figure 6c exhibits a clear enhancement in responsivity, with the Sb_2_S_3_-coated device reaching a peak of 0.47 A/W at 750 nm, compared to around 0.60 A/W at 810 nm for the pristine device. This improvement is attributed to the enhanced photon-to-electron conversion efficiency and better charge carrier transport [54]. In the meantime, the detectivity, as shown in Figure 6d, exhibits a similar pattern. The baseline device reaches only 5.4 × 10^15^ Jones at 750 nm, while the Sb_2_S_3_-based structure peaks at a detectivity of 1.05 × 10^17^ Jones at 810 nm. This improvement is around a two-order magnitude increase in detectivity, which is mainly due to higher photocurrent and suppressed dark current, due to the presence of an additional built-in potential at the Ba_3_SbI_9_/Sb_2_S_3_ interface [97]. At wavelengths beyond 890 nm, both devices experience a rapid drop in detectivity, caused by insufficient photon energy to generate charge carriers. These observations confirm the strong performance advantage of the Sb_2_S_3_ layer in enhancing the photodetector performance parameters.

#### 3.2.3. The Influence of Ba_3_SbI_3_ (Absorber) Layer Width on the Photodetector

The thickness of Ba_3_SbI_3_ layer is a crucial factor in determining the performance of photodetectors. In such applications, absorber layer thickness directly impacts light absorption. If the layer is too thin, it may not capture enough incident photons, leading to reduced photocurrent. Conversely, overly thick layers can increase the probability of charge carrier recombination before they reach the electrodes, ultimately decreasing the device’s efficiency [69]. The optimal thickness must balance photon absorption, efficient charge transport, photogeneration, and recombination. In this section, we examine how variations in absorber layer thickness influence the performance of the Ba_3_SbI_3_-based photodetector. The thickness is tuned between 0.4 µm and 1.4 µm, whereas the doping concentration and defect density are fixed at 1 × 10^14^ cm^−3^ and 1 × 10^17^ cm^−3^, respectively.

**Figure 7:**
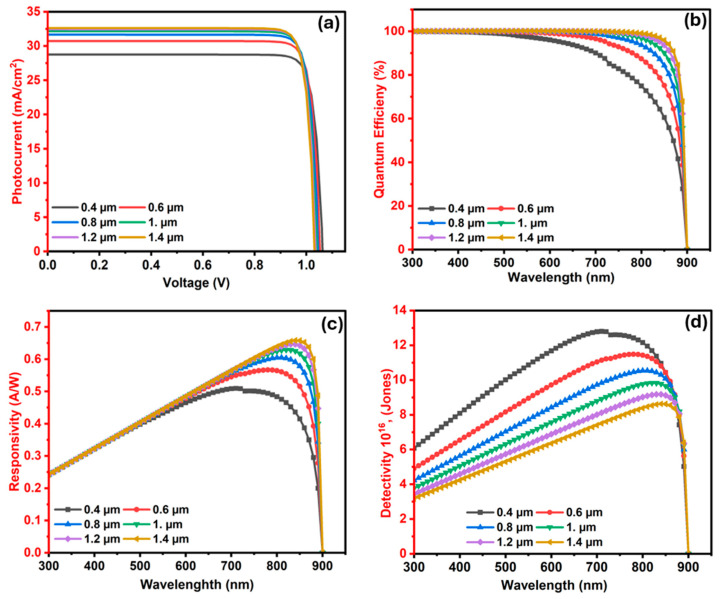
The effect of the Ba_3_SbI_3_ layer width on photodetector characteristics: (**a**) (J–V), (**b**) QE, (**c**) responsivity, and (**d**) detectivity.

As manifested in Figure 7a, it can be seen that at an absorber thickness of 0.4 µm, the device realizes a Voc of 1.063 Volts and a Jsc of 28.747 mA/cm^2^. The limited thickness at this scale restricts light absorption, resulting in reduced photocarrier generation and a lower Jsc. However, the Voc remains relatively high due to minimal recombination effects [76]. As the thickness increases to 0.8 µm, Jsc significantly improves to 31.66 mA/cm^2^. At the same time, Voc diminishes slightly to 1.047 V. This enhancement in Jsc arises from improved photon absorption caused by a thicker Ba_3_SbI_3_ layer [97]. However, Voc experiences a minor decrease, which is attributed to increased bulk recombination and reverse saturation current [76]. Beyond 0.8 µm, Jsc starts to saturate, reaching 32.62 mA/cm^2^ at 1.4 µm, while Voc continues a minor decline to 1.0329 V. This behavior is caused by the growth in recombination losses with increasing Ba_3_SbI_3_ thickness. These observed trends are in good agreement with previously reported findings in the literature [74].

The QE spectra of the Ba_3_SbI_3_-based photodetector as a function of absorber layer thickness are illustrated in Figure 7b. In the short-wavelength region (300–500 nm), all devices exhibit nearly identical performance, with QE exceeding 99%, except for the 0.4 μm device, in which the QE remains at around 99% only up to 390 nm and then starts to drop significantly. Thickness-dependent variations become more pronounced in the long-wavelength region. For the 0.4 μm layer, QE decreases from 90.1% at 700 nm to 49.9% at 870 nm and 27.9% at 890 nm, before vanishing at 900 nm. At 0.8 μm, QE remains at 98.8% at 700 nm, 75.0% at 850 nm, and 47.9% at 890 nm, while at 1.0 μm, it improves to 99.6%, 90.1%, and 55.7% at the same wavelengths, respectively. Further extension is observed at 1.2 μm and 1.4 μm, where QE values reach 99.9% and 100% at 700 nm, and 62.3% and 67.9% at 890 nm. Increasing the thickness thus enhances the response, particularly in the longer-wavelength region, which can be attributed to stronger absorption enabled by the extinction coefficient [54]. Notably, the gain saturates beyond ~1.2 μm as the spectra nearly overlap, indicating that an absorber thickness in the range of 0.8–1.2 μm provides the optimum trade-off between broad spectral response and minimized recombination losses.

Figure 7c illustrates the responsivity variation with wavelength across different absorber thicknesses. It increases steadily with absorber thickness. At the fixed wavelength of 810 nm, the value grows from 0.476 A/W for a 0.4 µm layer to 0.646 A/W for a 1.4 µm layer, reflecting enhanced photon absorption and quantum efficiency in thicker films [51]. In addition to this monotonic improvement, the maximum responsivity shifts toward longer wavelengths as thickness increases. The peak, which is observed near 710 nm with 0.509 A/W for the thinnest film, gradually moves to 850 nm, where it reaches 0.658 A/W at 1.4 µm. Notably, beyond 850 nm the thinner films exhibit an earlier rapid decline in responsivity, while the thicker layers sustain higher values up to about 890 nm before also decreasing. At a thickness of 800 nm, the device achieved an optimal responsivity of 0.605 A/W at a wavelength of 810 nm. For all thicknesses, the responsivity vanishes beyond 900 nm, consistent with the absorption edge of Ba_3_SbI_3_, where photon energies fall below the bandgap and no longer contribute to carrier generation.

Figure 7d illustrates the influence of absorber thickness, ranging from 0.4 to 1.4 µm, on the detectivity of Ba_3_SbI_3_-based devices. The highest detectivity, exceeding 1.4 × 10^17^ Jones, is achieved for the thinnest layer at 0.4 µm. As the Ba_3_SbI_3_-based devices become thicker, a steady decline is observed in detectivity due to increased dark current, since detectivity is inversely proportional to the square root of dark current, as indicated by Equation (7). Although thicker absorbers improve long-wavelength absorption, they also introduce more recombination and noise, reducing sensitivity. This behavior is in agreement with previous reports, where detectivity consistently decreased as the absorber thickness increased [51].

A thickness of 0.8 µm offers the best compromise between absorption and carrier collection, yielding a Voc of 1.047 V, Jsc of 30.655 mA/cm^2^, responsivity of 0.605 A/W, and detectivity of 1.05 × 10^17^ Jones, and is therefore recommended as the optimal value for future device design.

#### 3.2.4. The Influence of Ba_3_SbI_3_ (Absorber) Layer Doping Level (N_A_) on the Photodetector Performance

This section rigorously probes the impact of doping levels in the Ba_3_SbI_3_ compound on the performance characteristics of photodetectors. Optimizing the performance of the photodetector heavily relies on the acceptor doping levels (N_A_) within the absorber’s layer (Ba_3_SbI_3_). A proper doping level is essential for tuning the carrier concentration, enhancing the depletion region width, and ensuring appropriate band alignment between the electron and hole transport layers [98]. To explore the influence of doping on the device parameters, the doping level is varied over a range from 1× 10^15^ to 1 × 10^19^ cm^−3^.

**Figure 8:**
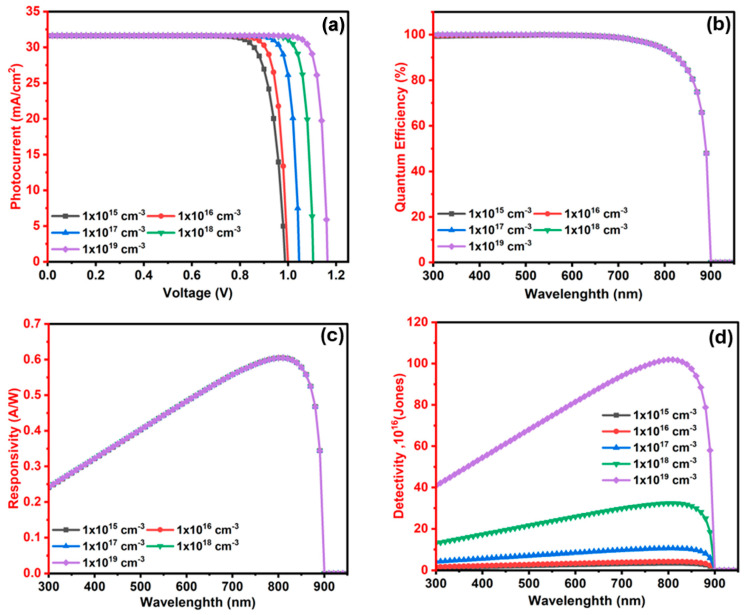
The effect of the Ba_3_SbI_3_ layer carrier concentration level on photodetector performance: (**a**) (J–V), (**b**) QE, (**c**) responsivity, and (**d**) detectivity.

Figure 8a reveals a pronounced increase in Voc from 0.988 to 1.165 Volt as the doping level of Ba_3_SbI_3_ rises to 1 × 10^19^ cm^−3^. The enhancement in Voc is attributed to the stronger built-in electric field formed at the junction, which improves charge separation and reduces carrier recombination [99]. In contrast, Jsc shows only slight variations, beginning at 31.62 mA·cm^−2^ at 1 × 10^15^ cm^−3^, increasing slightly to 31.66 mA·cm^−2^ at 1 × 10^17^ cm^−3^, and then dropping slightly to 31.64 mA·cm^−2^ at 1 × 10^19^ cm^−3^. The small decline in the Jsc at higher doping indicates that the photogenerated carriers start to recombine in the absorber [100].

The QE, delineated in Figure 8b, exhibits a nearly constant value with respect to changes in the absorber doping level (N_A_). It stays near 100% over the spectral range from 300 nm to 600 nm. After 550 nm, it progressively diminishes and reaches zero at around 900 nm, reflecting the material’s optical bandgap limit, beyond which photons do not possess sufficient energy for absorption [100]. This cutoff behavior confirms the optical absorption edge of Ba_3_SbI_3_ and underscores its strong potential for applications in visible and near-infrared optoelectronic devices.

Likewise, the responsivity exhibits a similar trend to the QE, as shown in Figure 8c. It remains largely unaffected by variations in doping levels, maintaining consistent values across the entire doping range. At a wavelength of 300 nm, all doping levels show a responsivity of approximately 0.24 A/W. The device achieves optimal photodetector performance with a peak responsivity of around 0.605 A/W at 810 nm. Beyond this point, a noticeable decline occurs, with a significant drop around 880 nm, and it eventually approaches zero near 900 nm. This observed behavior is in good agreement with previously reported results [76]. The detectivity, as shown in Figure 8d, improves significantly with higher doping, rising from 1.36 × 10^16^ Jones at 1 × 10^15^ cm^−3^ to above 1.02 × 10^18^ Jones at 1 × 10^19^ cm^−3^ at 810 nm. This enhancement is attributed to the reduction in dark current, as elevated doping strengthens the built-in electric field, enabling more efficient carrier extraction and suppressed recombination, which are critical for achieving high detectivity [101]. While the doping concentration of the Ba_3_SbI_3_ absorber has minimal impact on photogeneration properties such as Jsc, quantum efficiency, and responsivity, it plays a vital role in enhancing Voc and detectivity. Therefore, a moderate doping level offers the best balance, and the optimal device with a doping concentration of 1 × 10^17^ cm^−3^ delivers the most favorable performance, with parameters of Voc = 1.047 V, Jsc = 30.655 mA·cm^−2^, responsivity of 0.605 A /W, and D^*^ = 1.05 × 10^17^ Jones at 810 nm.

#### 3.2.5. The Influence of Ba_3_SbI_3_ (Absorber) Layer Defects (N_t_) on the Photodetector

Photodetector performance is also defect-level-dependent. This defect can be crystal defects and structural imperfections, which serve as recombination or trapping centers for photogenerated carriers, significantly affecting the operational performance of the photodetector [102,103]. To evaluate the effect of defect levels on Ba_3_SbI_3_-based photodetectors, a comprehensive analysis was performed across a defect spanning from 10^13^ to 10^17^ cm^−3^ while keeping the other features unchanged.

**Figure 9:**
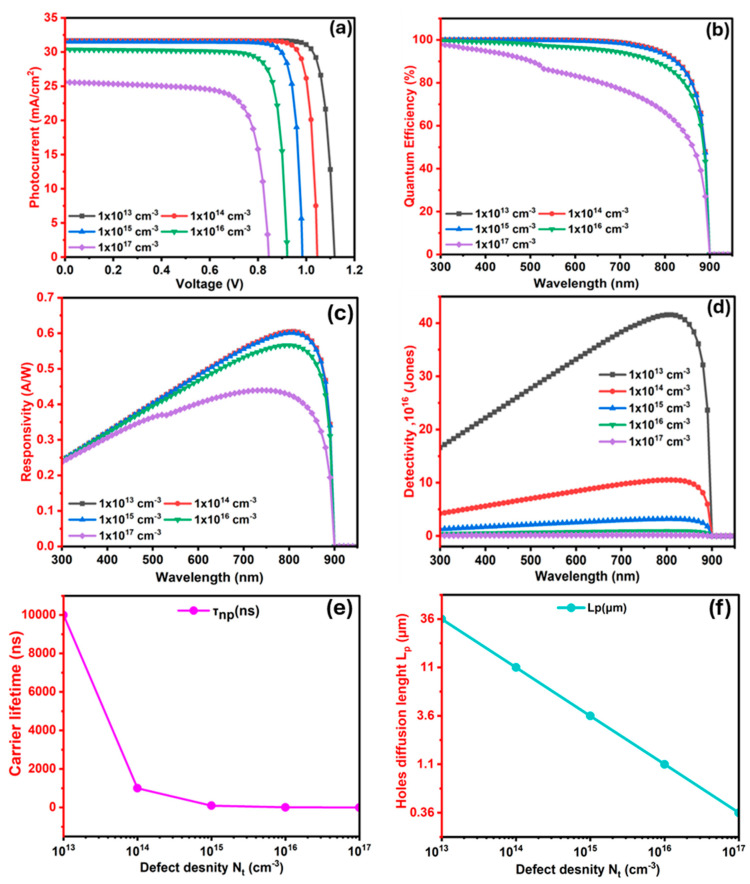
The influence of the Ba_3_SbI_3_ layer’s defect level on device features: (**a**) (J–V) curve; (**b**) QE; (**c**) responsivity as a function of Ba_3_SbI_3_’s defect level; (**d**) spectral detectivity as function of the Ba_3_SbI_3_ defects; (**e**) carrier lifetime for electron- and hole-dependent Ba_3_SbI_3_ defect level; (**f**) hole diffusion length-dependent Ba_3_SbI_3_ defect level.

As shown in Figure 9a, Voc significantly diminishes from 1.22 V to 0.85 V as the defect density increases. Meanwhile, Jsc is shown to decrease from 31.67 to 25.59 mA/cm^2^. Increasing defect density harms device performance by introducing trap states within the bandgap, which enhance Shockley–Read–Hall (SRH) recombination. This elevated recombination reduces carrier lifetime and ultimately decreases the efficiency, stability, and overall reliability of the device [52].

The QE is strongly influenced by defect concentration of the absorber, as shown in Figure 9b. At low defect densities, ranging from 1 × 10^13^ to 1 × 10^14^ cm^−3^, QE remains nearly flat at above 98% across 300–500 nm. It gradually decreases, reaching ~85 % at 850 nm and vanishing beyond 900 nm. With 1 × 10^15^ cm^−3^, QE stays above 95 % up to roughly 700 nm but declines more sharply at longer wavelengths, falling to ~75 % at 850 nm. At a defect level of 1 × 10^16^ cm^−3^, the drop becomes more pronounced beyond 700 nm, falling below 60% near 850 nm and approaching zero at 900 nm. A further deterioration occurs at the highest defect density of 1 × 10^17^ cm^−3^, where QE falls below 95 % at 400 nm, drops to ~50% at 800 nm, and vanishes past 880 nm. This degradation arises from reduced carrier lifetime and diffusion length due to SRH recombination, which increasingly suppresses carrier collection at higher defect densities, in line with earlier reports [54].

Figure 9c shows that the responsivity follows a similar trend to the QE. At 300 nm, all defect levels exhibit comparable responsivity of approximately 0.24 A/W, except for the device with a defect density of 1 × 10^17^ cm^−3^, which shows a slightly reduced value of 0.23 A/W. With increasing wavelength, responsivity progressively rises, reaching a peak of about 0.605 A/W at 810 nm for a defect density of 1 × 10^14^ cm^−3^. Beyond this point, responsivity decreases sharply and eventually falls to zero beyond 900 nm.

Detectivity trends, presented in Figure 9d, further emphasize the inverse relationship with defect density. The maximum detectivity decreases from 4.16 × 10^17^ Jones to 1.71 × 10^15^ Jones as the defect concentration increases from 10^13^ to 10^17^ cm^−3^. This decline in detectivity is linked to the reduction in Voc and the increase in dark current, both of which are related to detectivity as expressed in Equations (7) and (8). An increase in defect density reduces Voc, which directly influences the dark current; the latter is inversely related to detectivity [51]. Notably, the optimum performance is observed at a defect density of 1 × 10^14^ cm^−3^, where detectivity peaks at 1.05 × 10^17^ Jones at 810 nm.

To obtain deeper insight into the impact of absorber defect levels on photodetector performance, lifetime and diffusion length were analyzed. As shown in Figure 9e, a reduction in the bulk defect density (N_t_) of the absorber results in prolonged carrier lifetime and may increase the probability of generating more free charge carriers [104]. With an increase in carrier lifetime, charge separation and transport can be improved, allowing charges to be collected efficiently at both the front and back contacts of the photodetector device before they recombine, thereby enhancing the overall device efficiency [97].

Figure 9f illustrates the diffusion length for holes, which is calculated using Equation (18). In this expression, *D* denotes the diffusion coefficient, determined by Equation (19), and *τ* represents the photogeneration carrier lifetime of the bulk perovskite material, calculated using Equation (20). The symbol μ stands for the carrier mobility for electrons and holes, while $K_{B}$ is the Boltzmann constant. The *q* denotes the elementary charge, T is the temperature in Kelvin, σ represents the non-radiative recombination cross-section, and $V_{th}$ is the thermal voltage [104].

(18) $$L=\sqrt{D\times \tau }$$

(19) $$D=\frac{\;K_{B}*T}{q}\times \mu$$

(20) $$\tau =\frac{\;1}{\sigma \;V_{th}N_{t}}\times \mu$$

As observed from Figure 9f), a surge in the absorber’s defect density leads to a corresponding decrease in carrier diffusion length for electrons and holes. This behavior is attributed to enhanced non-radiative recombination processes, which reduce carrier lifetime and limit the distance that carriers can travel before recombination occur, as demonstrated by Equation (20). To balance the performance of the photodetector, a defect level of 1 × 10^14^ cm^−3^ for the absorber was chosen for further calculations.

#### 3.2.6. The Influence of In_2_S_3_ (ETL/Window) Layer Width on the Ba_3_SbI_3_ Photodetector

The ETL/window layer is critical for enhancing the overall performance of perovskite photodetectors. These layers facilitate the efficient extraction of photogenerated carriers from the active absorber and direct them toward the appropriate contacts [105]. Here, In_2_S_3_ is selected as the ETL due to its excellent stability and strong capability to collect and transport carriers [106]. To enhance the efficiency of photodetectors, the width of the ETL must always be smaller than that of the absorber layer. It should be moderately reduced to minimize interface resistance and allow more photons to reach the absorber. However, if the ETL is too thick, it can block incident light, reduce performance, and increase recombination. An optimal thickness is therefore required, thick enough to lower resistance and recombination, yet thin enough to enable efficient charge collection by the absorber [107].

**Figure 10:**
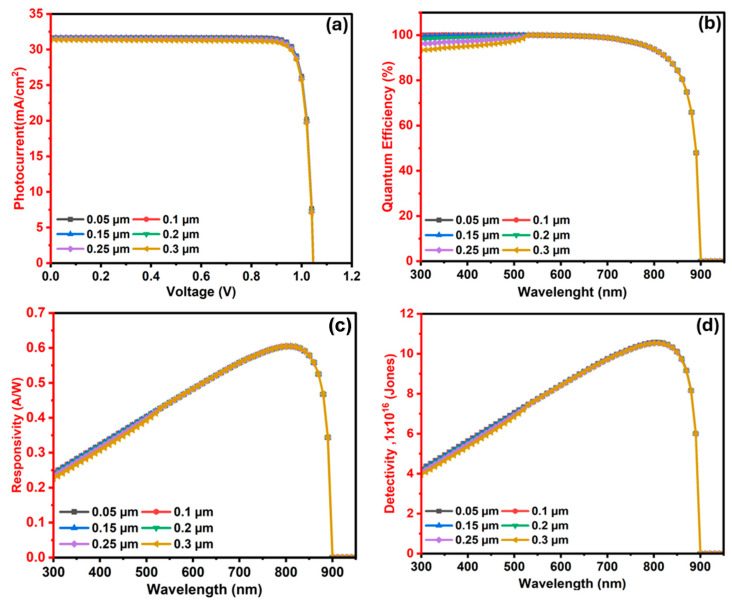
Dependence of Ba_3_SbI_9_-based photodetector properties on window-layer width: (**a**) J–V response; (**b**) QE spectrum; (**c**) responsivity; and (**d**) detectivity.

To examine its influence on the PD parameters, the In_2_S_3_ layer width was customized in the span from 0.05 to 0.3 μm. Figure 10a shows the impact of In_2_S_3_ ETL layer width on the operation of the Ba_3_SbI_3_-based photodetector. While Voc remains nearly stable at around 1.047 Volt, Jsc sinks from 31.65 to 31.35 mA/cm^2^ with the increase in In_2_S_3_ width. This reduction in Jsc is primarily due to enhanced parasitic absorption within the thicker window layer, which limits the penetration of high-energy photons into the absorber layer, thereby reducing the generated photocurrent [108].

QE as a function of wavelength for tuning In_2_S_3_ width is indicated in Figure 10b. As the In_2_S_3_ width changes in the range from 0.05 to 0.3 μm, QE remains relatively stable and close to 100% across the visible spectrum, particularly in the range of 550–750 nm. However, at shorter wavelengths spanning from 300 to 550 nm, a noticeable fluctuation in QE is observed with increasing thickness. This reduction is associated with window gain, where the window layer absorbs part of the incident high-energy photons instead of allowing them to reach the absorber layer. A thicker buffer layer also promotes higher recombination losses, as more carriers are trapped or lost before contributing to current, which lowers both Jsc and QE [76]. Beyond 900 nm, QE drops sharply to zero, as the photodetector becomes transparent to longer wavelengths due to the bandgap edge.

Figure 10c shows the responsivity of the Ba_3_SbI_3_ photodetector, which closely imitates the QE trend. The highest responsivity, approaching 0.605 A/W, is recorded near 810 nm by the thinner In_2_S_3_ layer (0.1 µm), and it decreases slightly as the width rises. This behavior is ascribed to reduced light transmission and increased recombination at greater thickness. The detectivity-dependent wavelength for In_2_S_3_ layer widths ranging from 0.05 to 0.3 μm is visualized in Figure 10d. It increases marginally with wavelength, reaching a maximum of approximately 1.05 × 10^17^ Jones near 810 nm. However, it decreases insignificantly in the shorter wavelength region (300–550 nm) with increasing window width. This behavior may be attributed to variations in QE and responsivity within the same wavelength range. This observation signifies that a thinner buffer layer is favorable for achieving higher photodetector sensitivity due to enhanced optical and electrical performance. Therefore, from these observations, it is noted that the photodetector with a thickness of 0.1 μm attains the optimal results. The overall behavior of this buffer on the cell was also observed in previous work on photodetectors [76].

#### 3.2.7. The Influence of In_2_S_3_ (ETL/Window) Layer Donor Level (N_D_) on the Ba_3_SbI_3_ Photodetector

**Figure 11:**
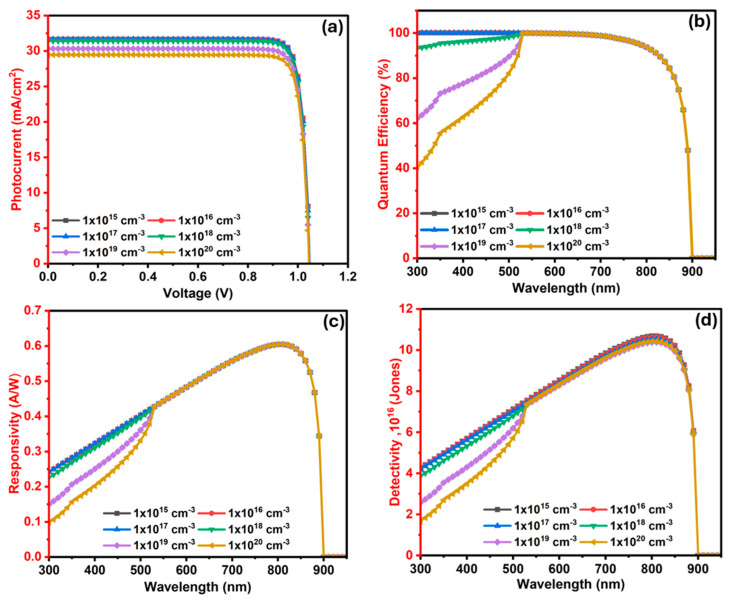
Dependence of Ba_3_SbI_9_-based photodetector properties on window-layer doping: (**a**) J–V response; (**b**) QE spectrum; (**c**) responsivity; and (**d**) detectivity.

Figure 11a illustrates the influence of the donor level (N_D_) in the In_2_S_3_ window layer on the J–V features of the PD. The donor level N_D_ is varied within a range of 1 × 10^15^–1 × 10^20^ cm^−3^. In this range, Voc seems to be unaffected at 1.047 Volt. Concurrently, Jsc exhibits an insignificant reduction from 31.66 to 29.46 mA·cm^−2^. As the doping concentration rises, the probability of electron–hole pair (EHP) recombination also increases, leading to a reduction in photocurrent [76].

Figure 11b shows QE as a function of wavelength for different doping concentrations of the In_2_S_3_ layer. For low doping levels up to 1 × 10^17^ cm^−3^, the QE remains nearly ideal, remaining at 100% in the spectral range of 300–530 nm. However, at higher doping concentrations 1 × 10^18^ cm^−3^ and above, the QE begins to deteriorate, particularly in the ultraviolet (UV) region. This is caused by increased recombination in the window gain [54]. Notably, in the visible and near-infrared range (550–850 nm), QE remains high, indicating effective photogeneration and collection in the absorber layer. Beyond 900 nm, a sharp drop in QE is observed across all doping levels, signifying the wavelength cutoff point of the photodetector.

Figure 11c presents the responsivity of the device for different doping levels of the In_2_S_3_ layer. At low doping from 1 × 10^15^–1 × 10^17^ cm^−3^, responsivity remains steady over a broad spectral range from 300 to 850 nm, reaching its peak value at 0.605 A/W at 810 nm. As donner doping volume increases, responsivity significantly declines in the UV region. For instance, at 300 nm, responsivity drops from 0.24 A/W to 0.098 A/W when the doping level increases from 1 × 10^15^ to 1 × 10^20^ cm^−3^. This trend mirrors the QE behavior and is attributed to enhanced carrier recombination and optical losses in the heavily doped window layer. Nonetheless, in the visible region, responsivity remains reasonably high even at higher doping levels.

**Figure 12:**
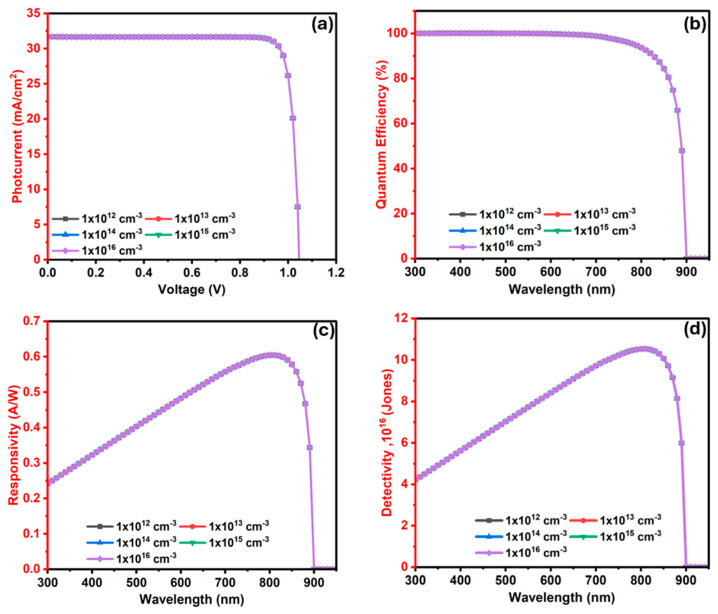
Dependence of Ba_3_SbI_9_-based photodetector properties on window-layer bulk defects: (**a**) J–V response; (**b**) QE spectrum; (**c**) responsivity; and (**d**) detectivity.

Figure 12d depicts the impact of the donor doping level of the In_2_S_3_ layer on the detectivity curves as the doping varies from 10^15^ to 10^20^ cm^−3^. As can be seen, the value of detectivity is slightly attenuated at higher doping concentrations. In the short-wavelength region (300–530 nm), detectivity shows a pronounced reduction with doping. At 300 nm, it is 4.28 × 10^16^ Jones for 10^15^ cm^−3^, decreasing to 2.58 × 10^16^ Jones at 10^19^ cm^−3^ and further down to 1.69 × 10^16^ Jones at 10^20^ cm^−3^. This is likely due to the reduced EQE and responsivity in the same spectral range, associated with the ETL window gain. As the wavelength increases, detectivity rises and reaches its maximum near 810 nm, where the value is 1.05 × 10^17^ Jones at 10^17^ cm^−3^, slightly decreasing to 1.04 × 10^17^ Jones at 10^20^ cm^−3^. Beyond 820 nm, detectivity rapidly falls by several orders of magnitude, approaching a negligible value after 900 nm.

This overall finding indicates that moderate carrier concentration levels between 1 × 10^16^ and 1 × 10^17^ cm^−3^ in the In_2_S_3_ window layer offer an optimal balance between electrical conductivity and minimal recombination loss. Higher doping concentrations beyond 1 × 10^18^ cm^−3^ degrade the device’s performance metrics, including Jsc, Voc, QE, responsivity, and detectivity. Thus, precise control over the window layer doner doping is critical for maximizing the efficiency and sensitivity of In_2_S_3_-based photodetectors across the UV–visible–NIR spectrum. This is why a carrier concentration level of 1 × 10^17^ cm^−3^ is selected as an optimum for the window layer In_2_S_3_ for further optimization.

#### 3.2.8. The Influence of In_2_S_3_ (ETL/Window) Layer Defects (N_t_) on the Ba_3_SbI_3_ Photodetector

This section scrutinizes how the defect level $N_{t}$ in the In_2_S_3_ window layer influences the PD parameters. To investigate this effect, the $N_{t}$ in the In_2_S_3_ was varied over a range from 1 × 10^12^ to 1 × 10^16^ cm^−3^ to discover its impact on overall device characteristics.

As shown in Figure 12a, the short-circuit current density (Jsc) stays nearly constant at approximately 31.65 mA·cm^−2^, even with a four-order-of-magnitude increase in defect density. Similarly, Voc remains constant at 1.047 V throughout the entire defect range. Likewise, QE, as indicated in Figure 12b, follows a consistent trend with fluctuation in the In_2_S_3_ defect level, stays close to 100% in the visible region, and begins to decline beyond 850 nm and drops to nil after 900 nm.

The magnitude of responsivity, illustrated in Figure 12c, peaks at 0.605 A/W at 810 nm and displays flat behavior with the increase in flaw density. Apart from responsivity, the detectivity, shown in Figure 12d, maintains a maximum of 1.05 × 10^17^ Jones in the NIR region, highlighting excellent infrared sensitivity. Since the carrier diffusion length and lifetime are much larger than the thickness of the In_2_S_3_ layer, increasing its defects does not significantly affect the overall device performance [109]. The defect tolerance can be further understood in terms of the carrier lifetime and diffusion length in the In_2_S_3_ window layer. These relationships are defined by Equations (18)–(20) above. The stability of device performance over a wide range of $N_{t}$ values implies that τ and L_n,p_ remain large enough to allow carriers to reach the junction before recombining, preserving high quantum efficiency and signal integrity.

### 3.3. Effect of Working Temperature on Photodetector Performance

Some photodetectors may be used in applications where the temperature is higher than room temperature and must operate under unstable conditions where the temperature varies. This variation may affect device performance [51]. Therefore, photodetectors must maintain high performance despite thermal fluctuations to ensure reliable operation under changing environmental conditions. In this work, a Ba_3_SbI_3_-based photodetector was characterized between 200 K and 450 K, and its temperature-dependent behavior was analyzed.

**Figure 13:**
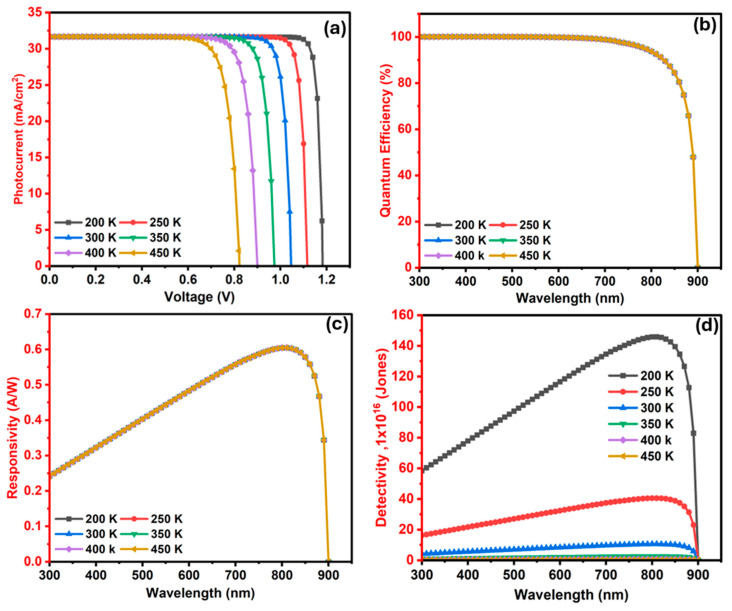
The Ba_3_SbI_3_-based photodetector performance fluctuation-dependent operating temperature (T): (**a**) (J–V) characteristics, (**b**) QE (**c**) responsivity, and (**d**) detectivity.

The J–V characteristics of the photodetector at different temperatures are shown in Figure 13a. It is observed that Voc experiences a remarkable drop from 1.1836 V at 200 K to 0.82 V at 450 K. In contrast, Jsc increases only slightly from 31.64 mA/cm^2^ to 31.66 mA/cm^2^ across the temperature range. The deterioration in Voc is primarily caused by bandgap narrowing at elevated temperatures, which enhances carrier recombination, increases defect states, and shortens diffusion lengths, thereby limiting the attainable Voc. This reduction in the bandgap also accounts for the slight increase in Jsc at higher temperatures. This pattern is also observed in other semiconductor-based photodetectors, highlighting the intrinsic thermal limitations of such devices [52]. Figure 13b delimits the QE dependence on working temperature. The QE shows negligible temperature dependence, remaining close to 100% from 300 to about 550 nm and then gradually decreasing to 97% around 750 nm. A sharper drop is observed in the NIR, reaching 66% at 880 nm and becoming almost zero near 900 nm.

Similarly, the responsivity exhibits remarkable stability, attaining a peak of 0.605 A·W^−1^ at 810 nm as delineated in Figure 13c. However, it is significantly affected in terms of detectivity, showing an apparent sensitivity to temperature. It drops from around 1.4 × 10^18^ Jones at 200 K to 1.4 × 10^15^ Jones at 450 K as depicted in Figure 13d. Such deterioration is driven by the growth in reverse saturation current with the surge in temperature, which impairs detectivity [110]. Despite this increase in temperature, the photodetector still exhibits competitive responsivity and detectivity even at a high temperature of 450 K, which may be useful for applications operating in uncooled environments. Hence, while photocurrent, QE, and responsivity remain almost unaffected by temperature, Voc and detectivity degrade severely, making 300 K the optimum operating point for balanced photodetector performance.

#### 3.3.1. Optimized Device Properties

Table 5 expounds the key performance parameters of the optimized devices with and without the incorporation of the (Sb_2_S_3_) layer, clearly demonstrating improvements across all metrics. The optimized device elevates the magnitude of Voc from 0.9017 volts to 1.047 volts, while Jsc rises from 26.8 mA/cm^2^ to 31.66 mA/cm^2^. In addition, the spectral response exhibits a red shift from 750 nm to 810 nm, thereby extending absorption into the near-infrared region. The responsivity improves from 0.51 A/W to 0.605 A/W, and the specific detectivity (D*) shows a considerable enhancement from 5.41 × 10^15^ to 1.05 × 10^17^ Jones, corresponding to nearly a 2-fold increase. Collectively, these results confirm that the integration of the Sb_2_S_3_ layer significantly enhances the photodetection efficiency of the device.

#### 3.3.2. Performance Comparison

Table 6 provides an in-depth comparison of the photoresponse properties obtained in this study with those reported in earlier experimental and simulation-based investigations. The experimental device with the MoS_2_/PtS structure demonstrated good responsivity but relied on chemical vapor deposition and electron-beam evaporation for fabrication of the device, making its production more expensive. Regarding the MXene/MAPbI_3_ device, although it has demonstrated good performance, it incorporates Pb material, which is a highly toxic element, raising significant concerns for human health and environmental safety. BaZrS_3_-based photodetectors showed limited responsivity and required sulfurization at elevated temperatures (~1050 °C), thereby restricting their scalability. Similarly, the TaC:Cu/SiC device exhibited relatively low detectivity despite the adoption of co-sputtering techniques.

In simulation-based studies, Ge_2_Sb_2_Te_5_ and graphene/GaAs heterostructures achieved notable photoresponse but relied on scarce and costly elements such as Ge, Te, Ga, and As, limiting their practical applicability. The p–Mg_2_Si/i–Mg_2_Si/n–Si stack simulated using TCAD Silvaco demonstrated a broadband response but only moderate performance, while the p-MoS_2_ architecture (SCAPS-1D) yielded modest responsivity and detectivity under external bias rather than self-powered conditions. Several SCAPS-1D-simulated structures, including CdS/Cu_2_ZnGeSe_4_/ZnTe and PbS/TiS_3_, operated in powered mode but incorporated highly toxic elements such as Cd and Pb. Other proposed material combinations, such as In_2_S_3_/BeSiP_2_/MoS_2_, introduced a rare element (Be). Although heterostructures based on n-WS_2_/Ag_3_CuS_2_/BaSi_2_ and ZnSe/SrHfSe_3_/AgCuS avoided highly toxic elements, their sensitivity was primarily restricted to shorter wavelengths, with limited response beyond 1 μm.

Here, this design presents an n-In_2_S_3_/p-Ba_3_SbI_3_/p^+^-Sb_2_S_3_ heterostructure that exhibits high responsivity and detectivity at 810 nm, while utilizing elementally abundant and environmentally benign chalcogenides, highlighting its potential as a sustainable and cost-effective material system for next-generation photodetectors.

## 4. Conclusions

This work conducted a comprehensive theoretical study on the novel lead-free inorganic perovskite Ba_3_SbI_3_, highlighting its suitability for photodetector applications. Employing density functional theory (DFT) with both PBE and HSE06 methods, we investigated the structural, electronic, optical, and mechanical properties of Ba_3_SbI_3_. The results confirmed that Ba_3_SbI_3_ is a mechanically stable material with a direct bandgap of 0.78 eV (PBE) and 1.60 eV (HSE06), along with strong optical absorption and favorable electronic characteristics. These attributes point to its high potential for use in light-harvesting and photodetection devices. The performance of a proposed device architecture, Al/FTO/In_2_S_3_/Ba_3_SbI_3_/Sb_2_S_3_/Ni, was evaluated using SCAPS-1D simulations, demonstrating impressive optoelectronic metrics including high Jsc, Voc, responsivity, and detectivity. The physical attributes of each layer of the device, such as width, doping level, and defect level, were altered to attain optimal performance. The optimized device yielded a Voc of 1.047 Volt, a Jsc of 31.65 mA/cm^2^, a responsivity of 0.605 A/W, and a detectivity of 1.05 × 10^17^ Jones at 810 nm. Importantly, even in the absence of the Sb_2_S_3_ layer, the photodetector maintained considerable efficiency, indicating the robustness and flexibility of the device design. Overall, this study establishes Ba_3_SbI_3_ as a promising, environmentally benign, and cost-effective material which could be an alternative material to lead-based perovskites. Its excellent structural and optoelectronic properties, combined with the demonstrated high-performance photodetector simulations, position Ba_3_SbI_3_ as a strong candidate for future applications in self-powered photodetectors and next-generation photonic technologies. Further experimental validation is encouraged to realize its practical implementation in real-world devices.

## Figures and Tables

**Table 1:** Lattice constant, optimized cell volume, total energy, and density of Ba_3_SbI_3_ as obtained in this work, compared with previous studies based on density functional theory (DFT) calculations.

Lattice Constant *a*(Å)	Optimum Volume (Å^3^)	Final Enthalpy	Density (Amu Å^−3^)	References
7.05	350.4026	3199.64855	2.609	This work
7.05	350.403	−3199.64	-	[64]
7.055	351.133	-	-	[49]
7.07	2387.1922	-	-	[46]
7.08	-	-	-	[47]
7.04	350.091	−3199.65	2.612	[50]
7.05	330	-	-	[48]

**Table 2:** Calculated tolerance factor of Ba_3_SbI_3_ compound.

Parameter	r_A_	r_sb_	r_X_	Tolerance Factor
Ba_3_SbI_3_	1.42	0.71	2.2	0.865

**Table 3:** Calculated elastic constants and mechanical parameters of Ba_3_SbI_3_.

Parameter	Our Calc.	Other Theo.
C11 (GPa)	53.366	53.817 ^a^, 54.067 ^b^
C12 (GPa)	6.5767	7.0199 ^a^, 7.0366 ^b^
C44 (GPa)	8.6655	8.6732 ^a^, 8.6379 ^b^
Bulk modulus B (GPa)	22.17	22.62 ^a^, 22.71 ^b^
Shear modulus G (GPa)	13.07	13.08 ^a^, 13.07 ^b^
Young’s modulus E (GPa)	32.77	32.89 ^a^, 32.91 ^b^
Anisotropy factor A	0.372	
Universal anisotropy factor A_U_	1.28	1.28 ^a^, 1.31 ^b^
Poisson’s ratio v	0.254	0.235 ^a^, 0.258 ^b^
Pugh’s ratio B/G	1.70	1.729 ^a^, 1.737 ^b^

^a^ [64], ^b^ [50].

**Table 4:** Tabulates the bandgap, energy cutoff, and k-point grid parameters.

Compound	Bandgap (eV)	E_Cut_ [eV]	k-Point	References
Ba_3_SbI_3_	0.78 (PBE) 1.602 (HSE)	500	8 × 8 × 8	This work
	1.512 (HSE)	550	10 × 10 × 10	[49]
	1.4 (HSE)	550	8 × 8 × 8	[48]
	1.38 (HSE)	-	-	[37]
	0.78 (PBE) 1.38 (HSE)	500	6 × 6 × 6	[64]
	0.856 (PBE) 1.384 (HSE)	410	6 × 6 × 6	[47]
	1.056 (PBE) 1.38 (HSE)	-	6 × 6 × 6	[46]
	0.78 (PBE)	400	6 × 6 × 6	[45]

**Table 5:** Photodetector characteristics with and without an Sb_2_S_3_ layer.

Structure	Voc	Jsc (mA/cm^2^)	Wavelength (nm)	Responsivity (A/W)	Detectivity (Jones)
In_2_S3/Ba_3_Sbi3_3_	0.9017	26.80	750	0.51	5.41 × 10^15^
In_2_S3/Ba_3_Sbi3_3_/Sb_2_S_3_	1.047	31.65	810	0.605	1.05 × 10^17^

**Table 6:** Comparison of the performance parameters of the Ba_3_SbI_9_-based photodetector developed in this work with those reported in the prior literature.

Structure	Types of Work	Software Used /Method	λ (nm)	Responsivity (A/W)	Detectivity (Jones)	Reference
MoS_2_/PtS	Experiment	E-beam evaporation + CVD	400	25.43	8.54 × 10^12^	[111]
MXene/MAPbI_3_	Experiment	Solution processed	525	1.70	7.0 × 10^11^	[112]
BaZrS_3_	Experiment	Solution + sulfurization at 1050 °C	405	46.5 × 10^−3^	-	[113]
TaC:Cu/4H Silicon Carbide	Experiment	Co-sputtering	405	1.66	2.69 × 10^8^	[10]
Ge_2_Sb_2_Te_5_	Simulation	Lumerical charge	1550	~48	-	[114]
Graphene/GaAs	Simulation	COMSOL Multiphysics	725	0.514	1.16 × 10^11^	[66]
p–Mg_2_Si/i–Mg_2_Si/n–Si	Simulation	TCAD Silvaco	400–1500	0.45	7.42 × 10^11^	[115]
p-MoS_2_	Simulation	SCAPS-1D	700	0.37	3.27 × 10^14^	[116]
CdS/p-Cu_2_ZnGeSe_4_/ p+-ZnTe	Simulation	SCAPS-1D	780	0.58	8.28 × 10^17^	[117]
PbS/TiS_3_	Simulation	SCAPD-1D	780	0.36	3.9 × 10^13^	[118]
n-ZnSe/p-TiSe_2_/p+-WSe2	Simulation	SCAPS-1D	920	0.670	12.90 × 10^14^	[103]
n-In2S3/p-BeSiP_2_/p+-MoS2	Simulation	SCAPS-1D	860	0.64	3.63 × 10^16^	[52]
n-WS2/p-Ag_3_CuS_2_/p+-BaSi2	Simulation	SCPDS-1D	1065	0.790	4.73 × 10^14^	[76]
ZnSe/p-SrHfSe_3_/p+-AgCuS	Simulation	SCAPS-1D	1100	0.850	2.26 × 10^14^	[51]
n-In_2_S_3_/p-Ba_3_SbI_3_/p+- Sb_2_S_3_	Simulation	SCAPS-1D	810	0.605	1.05 × 10^17^	This work

## Data Availability

The data supporting the findings of this study are available from the corresponding author upon reasonable request.

## Supplementary Materials

The following supporting information can be downloaded at https://www.mdpi.com/article/10.3390/nano15211656/s1. Table S1 lists the parameters employed in the simulation, while Table S2 reports the front and back contacts’ work function values. Table S3 summarizes the energy levels and band alignment of the FTO/In_2_S_3_/Ba_3_SbI_3_/Sb_2_S_3_/Ni structure. Table S4 provides the convergence summary for geometry optimization of Ba_3_SbI_3_. Table S5 and Table S6 present the lattice mismatch analysis of ETL and HTL layers, respectively, confirming the structural compatibility of In_2_S_3_ and Sb_2_S_3_ with Ba_3_SbI_3_. Table S7 details the convergence results for structural relaxation of Ba_3_SbI_3_, while Table S8 provides the parameters and constants used for calculating the noise equivalent power (NEP) of the photodetector. Table S9 compares photodetectors’ performance at different visible and near-infrared (NIR) wavelengths. Figure S1 demonstrates the effect of the Sb_2_S_3_-based back surface field (BSF) layer depth on device performance, including J–V characteristics, quantum efficiency (QE), responsivity, and detectivity. Figure S2 presents the influence of acceptors doping concentration and defect density in the BSF layer. Figure S3 illustrates the impact of interface defect density at the In_2_S_3_/Ba_3_SbI_3_ junction, while Figure S4 shows the corresponding effects at the Ba_3_SbI_3_/Sb_2_S_3_ heterojunction. Figure S5 depicts the effect of series and shunt resistance on the J–V characteristics, while Figure S6 presents the influence of resistance variations on overall device efficiency. References [119,120,121,122,123,124,125,126,127,128,129,130,131,132] are cited in the supplementary materials.

## Abbreviations

The following abbreviations are used in this manuscript:

Ba_3_SbI_3_	barium antimony iodide
CBM	conduction-band minimum
EC/EV	energy level of the conduction/valence band
ETL/HTL	electron/hole transport layer
Eg	bandgap
FTO	fluorine-doped tin oxide
InGaAs	indium gallium arsenide
In_2_S_3_	indium disulfide
JSC	short-circuit current density
J–V	current voltage
MAPbI_3_	methylammonium lead iodide
N_A_	acceptor density
N_t_	defect density
PDs	photodetectors
QE	quantum efficiency
Rs	series resistance
Rsh	shunt resistance
Sb_2_S_3_	antimony disulfide
SCAPS-1D	solar cell capacitance simulator one-dimension
SiC	silicon carbide
VBM	valence-band maximum
VOC	open-circuit voltage
