# Supplementary material for: Design and Optimization of Self-Powered Photodetector Using Lead-Free Halide Perovskite Ba3SbI3: Insights from DFT and SCAPS-1D
Source: Nanomaterials (Basel). 2025 Oct 30;15(21):1656. doi: 10.3390/nano15211656 (PMC12608577; doi:10.3390/nano15211656)
Supplement: Supplementary file 1 [file nanomaterials-15-01656-s001.zip › nanomaterials-3916273-supplementary.pdf]

Design and Optimization of Self-Powered Photodetector Using Lead-Free Halide Perovskite Ba_3SbI_3: Insights from DFT and SCAPS-1D

Salah Abdo [1], Ambali Alade Odebowale [1], Amer Abdulghani [1], Khalil As'ham [1], Yacine Djalab [2], Nicholas Kanizaj [1] and Andrey E. Miroshnichenko [1,*]

[1] School of Engineering and Technology, University of New South Wales at Canberra, Northcott Drive, Canberra, ACT 2610, Australia; s.abdo@unsw.edu.au (S.A.); a.odebowale@unsw.edu.au (A.A.O.); a.abdulghani@unsw.edu.au (A.A.); k.asham@unsw.edu.au (K.A.); n.kanizaj@unsw.edu.au (N.K.)

[2] Physico-Chemistry of Materials and Environment Laboratory, Physics Department, Ziane Achour University of Djelfa, Djelfa BP 3117, Algeria; yacine.djalab@univ-djelfa.dz

* Correspondence: andrey.miroshnichenko@unsw.edu.au; Tel.: +61-(2)-51145182

Table S1. Parameters employed in the simulation.

Structure	FTO [1]	In_2S_3 [2]	Ba_3SbI_3 [3]	Sb_2S_3[4, 5]
Thickness (μm)	0.05	0.1	0.8	0.2
Electron Affinity (eV)	4.5	4.4	4.25	3.8
Bandgap (eV)	3.6	2.35	1.384	1.7
Dielectric permittivity	10.0	13.5	6.02	7.08
Effective DOS at VB (cm^{-3})	1.800×10^{19}	1.800×10^{19}	1.164×10^{18}	1.0×10^{19}
Effective DOS at CB (cm^{-3})	2.0×10^{18}	2.20×10^{17}	9.613×10^{18}	2.8×10^{19}
Thermal velocity of holes (cm^{-1})	1.00×10^{7}	1.00×10^{7}	1.00×10^{7}	1.00×10^{7}
Thermal velocity of electron (cm^{-1})	1.00×10^{7}	1.00×10^{7}	1.00×10^{7}	1.00×10^{7}
Hole mobility ($cm^2\, cm^{-1}s^{-1}$)	2.00×10^{1}	2.50×10^{1}	5.00×10^{1}	1.00×10^{1}
Electron mobility ($cm^2\, cm^{-1}s^{-1}$)	1.00×10^{2}	1.00×10^{2}	5.00×10^{1}	9.80×10^{0}
Bulk defect density (cm^{-3})	1.00×10^{14}	1.00×10^{14}	1.00×10^{14}	1.00×10^{14}
Shallow uniform donor density N_D (cm^{-3})	1.00×10^{18}	1.00×10^{17}		0
Shallow uniform acceptor density N_A (cm^{-3})	0	0	1.00×10^{17}	1.00×10^{19}

Table S2. The interface defects parameters In_2S_3/Ba_3SbI_3 and Ba_3SbI_3/MoS_2.

Parameters (unit)	In_2S_3/Ba_3SbI_3	Ba_3SbI_3/MoS_2
Defect type	Neutral	Neutral
Capture cross section electrons (cm^2)	10^{-19}	10^{-19}
Capture cross section holes (cm^2)	10^{-19}	10^{-19}
Energy with respect to Reference (eV)	0.6	0.6
**Total** density (**cm^{-2}**)	10^{10}	10^{10}

Table S3. The contacts parameters applied in the simulation.

Contacts	Unit	Back contact parameters	Front contact parameters
Metal work function	eV	5.35 [6]	4.2 [7]
Surface recombination velocity of electrons	cm/s	1.00×10^5	1.00×10^5
Surface recombination velocity of holes	cm/s	1.00×10^7	1.00×10^7

1. Band alignment analysis of FTO/In_2S_3/Ba_3SbI_3/Sb_2S_3/Ni structure

This section presents the band alignment analysis for in the FTO/In_2S_3/Ba_3SbI_3/Sb_2S_3/Ni interfaces-based photodetector. The conduction band offset (CBO) and valence band offset (VBO) values are calculated using Eq. (S1) and Eq. (S2), respectively [8] .

At the In_2S_3/Ba_3SbI_3 interface, the conduction band offset (CBO) is given by:

$$\text{CBO} = x_{\text{absorber}} - x_{\text{ETL}} = -0.15 \text{ eV} \quad \text{(S1)}$$

Where the x_{absorber} and x_{ETL} are the electron for the absorber and ETL respectively. Since the ETL layer affinity is larger than the absorber layer affinity, a small cliff-like shape between the In_2S_3 and Ba_3SbI_3 layers is formed, which could suppress electron backflow.

The valence band offset (VBO) at the Ba_3SbI_3/Sb_2S_3 interface is determined using Eq. (S2), defined as:

$$\text{VBO} = \left(x_{\text{absorber}} - E_{g,\text{absorber}}\right) + \left(x_{\text{HTL}} + E_{g,\text{HTL}}\right) \quad \text{(S2)}$$

$$\text{VBO} = (4.25-1.384) + (3.80 + 1.70) = 5.634 - 5.50 = +\,0.134 \text{ eV.}$$

The Ba_3SbI_3/Sb_2S_3 interface exhibits a small positive valence band offset (VBO) of 0.134 eV, which help facilitating efficient hole transport from Ba_3SbI_3 into Sb_2S_3 while simultaneously suppressing interfacial recombination. Table S4 summarizes the bandgap alignment characteristics of the FTO/In_2S_3/Ba_3SbI_3/Sb_2S_3 heterojunction interfaces.

Table S4. Summary of the bandgap (Eg), electron affinity (χ), conduction band minimum (EC), valence band maximum (EV) for FTO, In_2S_3, Ba_3SbI_3, and Sb_2S_3 materials.

Material	Eg (eV)	χ (eV)	EC ≡ CBM (eV)	EV ≡ VBM (eV)
FTO	3.60	4.50	− 4.50	8.10
In_2S_3	2.35	4.40	− 4.40	6.75
Ba_3SbI_3	1.384	4.25	− 4.25	5.63
Sb_2S_3	1.70	3.80	− 3.80	5.50

2. Lattice mismatch analysis of $In_2S_3/Ba_3SbI_3/Sb_2S_3$ and other ETL/HTL interfaces

The selection of In_2S_3 as the electron transport layer (ETL) and Sb_2S_3 as the hole transport layer (HTL) is primarily based on their superior lattice compatibility and favourable interface quality with the Ba_3SbI_3 absorber. A good lattice match minimizes interfacial strain and suppresses defect formation, which are key factors in enhancing charge carrier mobility and overall device stability. The lattice mismatch can be calculated using the formula (S3) [9]:

$$\delta = \frac{2|a_s - a_e|}{a_s + a_e} \times 100 \qquad \text{(S3)}$$

where a_e is the lattice constant of the epitaxial layer and a_s is the lattice constant of the substrate. The lattice parameters used for the layers are derived from previously reported structural data. The calculated δ values for both β-In_2S_3 (tetragonal) and TiO_2 and ZnO with respect to Ba_3SbI_3, are summarized in Table S5. It is determined that the δ value at the β-In_2S_3/Ba_3SbI_3 junction is considerably lower than that at the TiO_2 and ZnO/Ba_3SbI_3 interface. In order for TiO_2 and ZnO to work efficiently with the Ba_3SbI_3 absorber, they would require large supercell arrangements to reduce δ to a realistic level, making them less efficiency for experimental fabrication and long-term stability.

Table S5. The lattice mismatch analysis summarized below demonstrates why β-In_2S_3 is the most suitable ETL compared to TiO_2 and ZnO.

Layer	a (Å)	b (Å)	c (Å)	Lattice mismatch δ (%)	Reference
Ba_3SbI_3 (absorber)	7.05	7.05	7.05		This work
In_2S_3 (β)	7.62	7.62	32.36	7.8 %	[10]
TiO_2	4.59	4.59	2.96	42.3 %	[9]
ZnO	3.242	3.242	5.188	74.0 %	[11]

Similarly, possible HTL (hole transport layer) materials were analysed to assess their structural compatibility with the Ba_3SbI_3 absorber. The calculated δ values for both Sb_2S and

MoS_2 with respect to Ba_3SbI_3 interfaces, are summarized in Table S6. The lattice mismatch results clearly indicate that Sb_2S_3 provides a much better interface match compared to MoS_2. Although Sb_2S_3 exhibits a noticeable lattice mismatch along one crystallographic axis (58.9%), it still offers a more stable and energetically favourable interface than MoS_2, which shows an extremely high mismatch of about 75.9%. Such a large mismatch in MoS_2 introduces significant lattice strain and misfit dislocations, which in turn increase nonradiative recombination and degrade interfacial charge extraction efficiency. Therefore, despite its partial mismatch, Sb_2S_3 remains the more favourable HTL candidate due to its better structural alignment, chemical stability, and compatibility with the Ba_3SbI_3 absorber. The superior structural coherence of tetragonal In_2S_3 with Ba_3SbI_3, combined with the acceptable interface quality of Sb_2S_3, contributes to enhanced charge transport, reduced recombination losses, and overall improved photodetector performance in the Al/FTO/In_2S_3/Ba_3SbI_3/Sb_2S_3/Ni device architecture.

Table S6. The lattice mismatch analysis summarized below of Sb_2S_3 layer compared with other HTLs including TiO_2 and ZnO.

Layer	a (Å)	b (Å)	c (Å)	Lattice mismatch δ (%)	Reference
Ba_3SbI_3 (absorber)	7.05	7.05	7.05		This work
Sb_2S_3 (orthorhombic)	11.311	3.839	11.223	58.9 % (b-axis)	[12]
MoS_2 (2H-phase)	3.190	3.190	14.879	75.9 %	[13]

3. Relaxing the B_3SbI_3 compound

The wavefunction cutoff energy and the Monkhorst–Pack k-point grid were independently optimized by testing multiple values to minimize the total energy difference between the initial and final volumes. The cutoff energy and the Monkhorst–Pack k-point that produced the smallest deviation were selected, yielding an optimal cutoff energy of 500 eV and a k-point mesh of 8 × 8 × 8.The optimized cutoff energy and k-point mesh were subsequently employed to relax the Ba_3SbI_3 structure. This procedure enabled accurate minimization of the lattice constants, atomic positions, and total energy, ensuring that the equilibrium geometry was achieved. The resulting relaxed structure provided a reliable foundation for the subsequent electronic and optical property calculations.

As summarized in Table S7, the convergence criteria were successfully met once all the convergence criteria were satisfied. This occurs when the energy change per atom fell well

below the threshold of 1.0×10^{-6} eV, the maximum stress decreased to 8.44×10^{-4} GPa, and the maximum forces were reduced to 4.61×10^{-4} eV/Å, confirming robust relaxation. The only criterion that lagged was the maximum atomic displacement, which exceeded the target in the early steps (2.20×10^{-4} Å and 1.86×10^{-4} Å versus the 1.0×10^{-4} Å limit. This condition was eventually satisfied in the third relaxation step and fully minimized to zero in step four, which indicates a complete convergence was achieved and no further iterations were necessary.

Table S7. Summary of structural relaxation results for Ba_3SbI_3 perovskite obtained using the GGA/BPE functional

Metric	Tolerance	Step 1	Step 2	Step 3	Step 4	Convergence Status
ΔE (eV/atom)	$\leq 1.0 \times 10^{-6}$	9.32×10^{-8} ✓	9.39×10^{-8} ✓	9.48×10^{-8} ✓	9.44×10^{-8} ✓	Converged
Max force (eV/Å)	$\leq 2.5 \times 10^{-3}$	7.28×10^{-4} ✓	4.61×10^{-4} ✓	4.61×10^{-4} ✓	4.61×10^{-4} ✓	Converged
Max stress (GPa)	$\leq 5.0 \times 10^{-3}$	5.31×10^{-4} ✓	3.43×10^{-3} ✓	3.43×10^{-3} ✓	8.44×10^{-4} ✓	Converged
Max displacement (Å)	$\leq 1.0 \times 10^{-4}$	2.20×10^{-4} ✕	1.86×10^{-4} ✕	1.00×10^{-4} ✓	0.00 ✓	Converged

4. Influence of Sb_2S_3 (BSF) layer

This section scrutinizes the impact of surface field (BSF) layer width, doping level, and defect density on various device parameters, including photocurrent density, QE, responsivity, and specific detectivity. The BSF is critical for improving photodetector efficiency. This layer is formed by introducing a heavy-doping thin film between the active layer and the back contact (Ni). The doping disparity between the absorber and the BSF layer generates a high electric field that reflects minority carriers back to the absorber layer. As a result, this electric field reduces recombination and contributes to higher current generation [14].

4.1 The fluctuation of the photodetector performance with the change in the Sb_2S_3 layer width

Figure S1 investigates the influence of the Sb_2S_3 (BSF) layer width on the performance metrics of the photodetector, within the range of 0.1 μm to 0.35 μm. As illustrated in Figure S1(a), increasing the BSF layer width has no significant effect on the Voc and Jsc. Both values remain constant at 1.047 V and 31.65 mA/cm², respectively. This negligible impact on the photodetector functionality could be ascribed to the minimal absorption of photons in the Sb_2S_3

layer [15].Similarly, the QE, as depicted in Figure S1(b), remains largely unaffected across BSF widths from 0.1 to 0.35 μm. It maintains nearly 100 % between 300 and 560 nm, after which it gradually decreases, reaching zero after 900 nm.

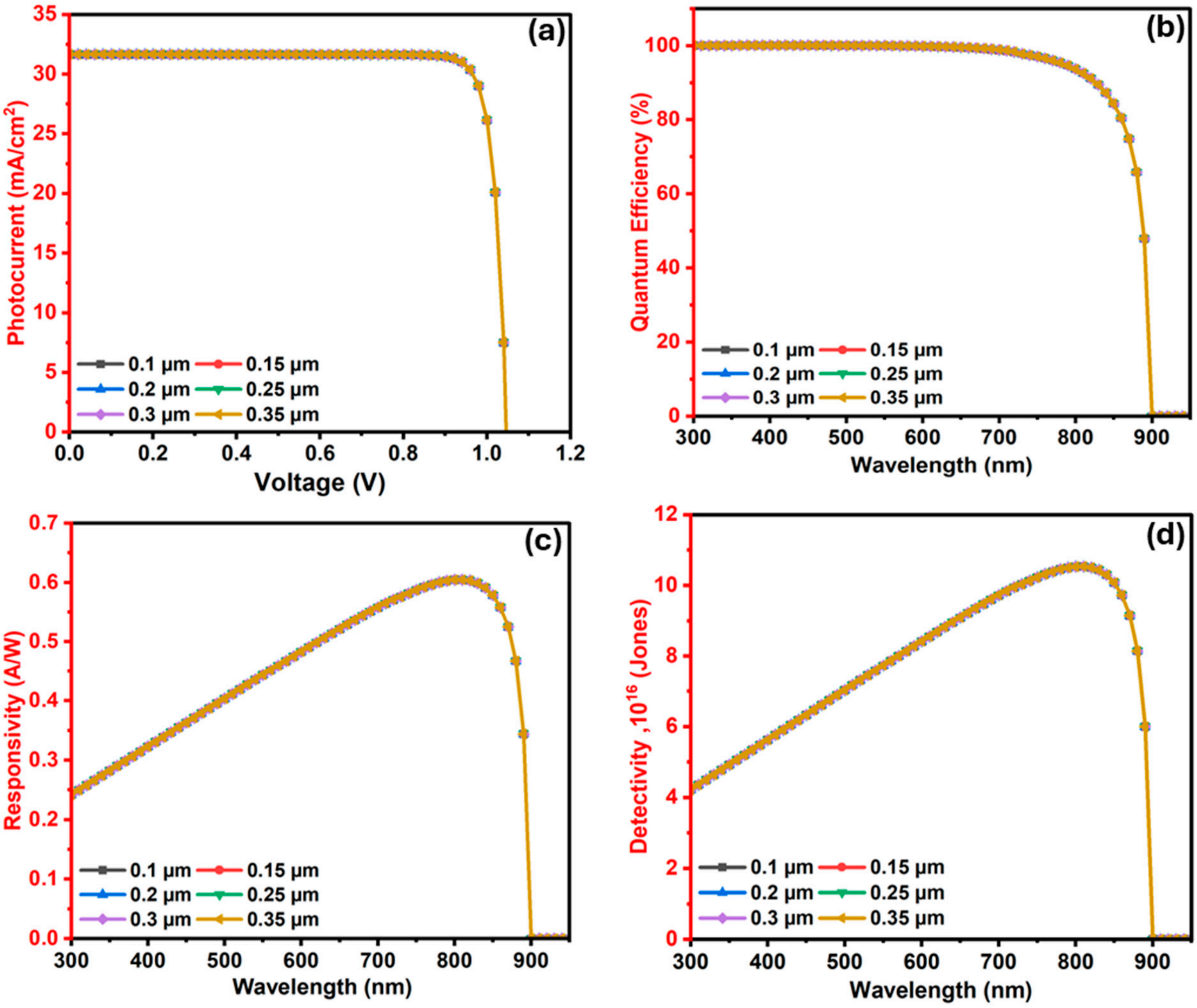

Figure S1. Effect of Sb_2S_3-based BSF width (0.1–0.35 μm) on the Ba_3SbI_3 photodetector (a) the I–V characteristics (b) QE (c) responsivity (d) detectivity.

Similarly, Figure S1(c) shows that responsivity remains stable as the BSF width varies in range from 0.1 to 0.35 μm. It starts at 0.22 $A \cdot W^{-1}$ at 300 nm. It displays its peak magnitude of 0.605 $A \cdot W^{-1}$ at a BSF width of 0.2 μm at 810 nm. After this value, it decays and drops to zero beyond 900 nm. As shown in Figure S1(d), the detectivity follows the same trend as the responsivity, remaining flat across all BSF widths. The diffusion length and lifetime of carriers greatly exceed the thickness of the Sb_2S_3 layer. Thus, variations in its thickness have only a minor influence on device performance [16]. The optimal value of the detectivity is observed at 0.2 μm, with 1.05×10^{17} Jones at 810 nm. However, beyond 900 nm, the detectivity decreases sharply, indicating reduced photoresponse in the longer-wavelength region

4.2 The Sb_2S_3 layer doping on the photodetector characteristics

This section thoroughly probes the effect of dopant level (N_A) variation in the Sb_2S_3 on photodetector functionality, with N_A swept from 1×10^{17} to 1×10^{21} cm^{-3}. As shown in Figure S2(a), both Voc and Jsc persevere roughly stable values at almost 1.047 V and 31.65 mA/cm², respectively, across all doping levels. The QE does not show any fluctuation by the surges of the dopant level, as indicated in Figure S2 (b), sustaining nearly 100 % from 300 nm to 560 nm, before sharply falling to zero beyond 900 nm.

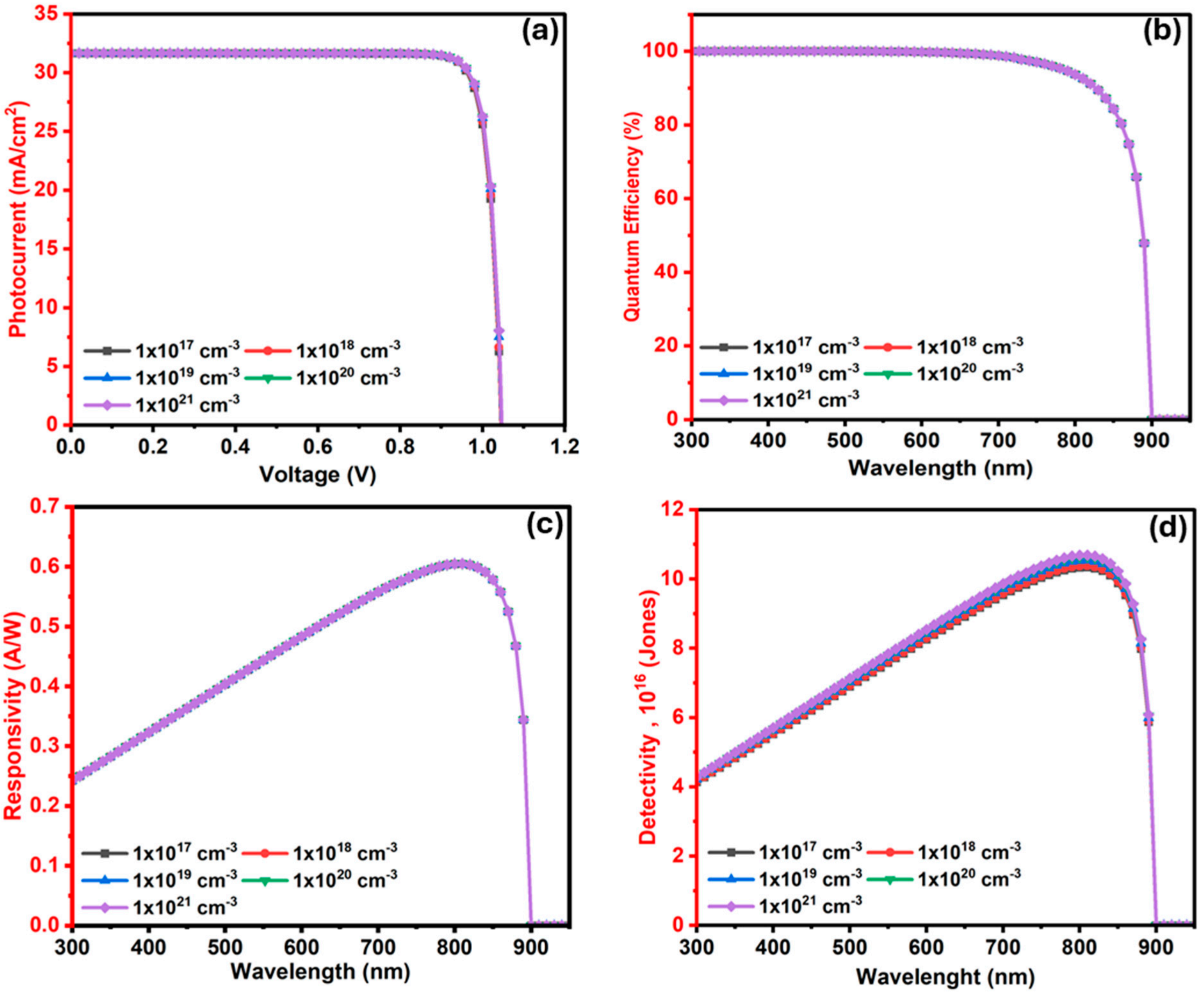

Figure S2. The effect of dopant level (N_A) in the Sb_2S_3-based BSF layer (ranging from 1×10^{17}-1×10^{21} cm^{-3}) on device performance (a) I-V characteristics (b) QE (c) responsiveness (d) detectivity.

In Figure S2(c), the responsivity mirrors the QE, exhibiting a consistent trend across all dopant levels, start at 0.24 $A\cdot W^{-1}$ at 300 nm and reaching its maximum value of 0.605 $A\cdot W^{-1}$

at 810 nm. The dependence of detectivity on the dopant level (N_A) in the Sb_2S_3 photodetector was examined across the range from $1×10^{17}$ to $1×10^{21}$ cm^{-3}.For doping in the $1 × 10^{17}$–$1 × 10^{18}$ cm^{-3} range, the detectivity lies between $4.1 × 10^{16}$ and $7.0 × 10^{16}$ Jones at wavelengths of 300–500 nm. Increasing the acceptor level further, from $1 × 10^{19}$ to $1 × 10^{21}$ cm^{-3}, leads to a climb in detectivity from $9.5 × 10^{16}$ Jones at 700 nm to nearly $1.07 × 10^{17}$ Jones at 810 nm. Beyond 900 nm, it falls rapidly, and the device becomes ineffective. The observed enhancement in device detectivity with increasing doping level is primarily ascribed to the suppression of dark current at higher dopant levels [6].

4.3 The Sb_2S_3 layer defects on the photodetector characteristics

In this section, the impact of varying the defect density in the Sb_2S_3 layer on overall device performance is comprehensively analysed. The defect density (N_t) is swapped systematically from $1×10^{12}$ to $1×10^{17}$ cm^{-3} to assess its influence on key performance parameters. This investigation aims to evaluate the device's tolerance to intrinsic material imperfections within the Sb_2S_3 layer and understand their implications for photodetection applications.

Figure S3 (a) shows the photocurrent density versus voltage characteristics. The device performance is flat and unaffected by the presence of defect states up to 10^{17} cm^{-3} level. For instance, the Jsc remains consistently around 31.65 mA/cm², and the Voc stays fixed at 1.047 V across all defect densities. Figure S3 (b) illustrates the QE dependent spectral wavelength for various defect levels. The QE remains impressively flat and close to 100 % in the spectrum extending from 300 nm to around 560 nm before dropping sharply beyond 900 nm. The responsivity, as shown in Figure S3(c), exhibits a similar trend to QE. It increases with wavelength and reaches a peak value of 0.605 A/W at 810 nm. The curves for all defect concentrations are nearly identical.

Figure S3 (d) presents the detectivity response over the wavelength range. The maximum detectivity is observed to be around $1.05 × 10^{17}$ Jones, corresponding to a defect level of $1 × 10^{17}$ cm^{-3} at 810 nm. Notably, this parameter remains nearly unchanged across all examined defect densities. This behaviour agrees well with previous work on photodetectors [15]. For the BSF layer, a thickness of 0.2 μm, a dopant level of $1 × 10^{19}$ cm^{-3}, and a defect density of $1 × 10^{14}$ cm^{-3} are considered optimal conditions for further optimization.

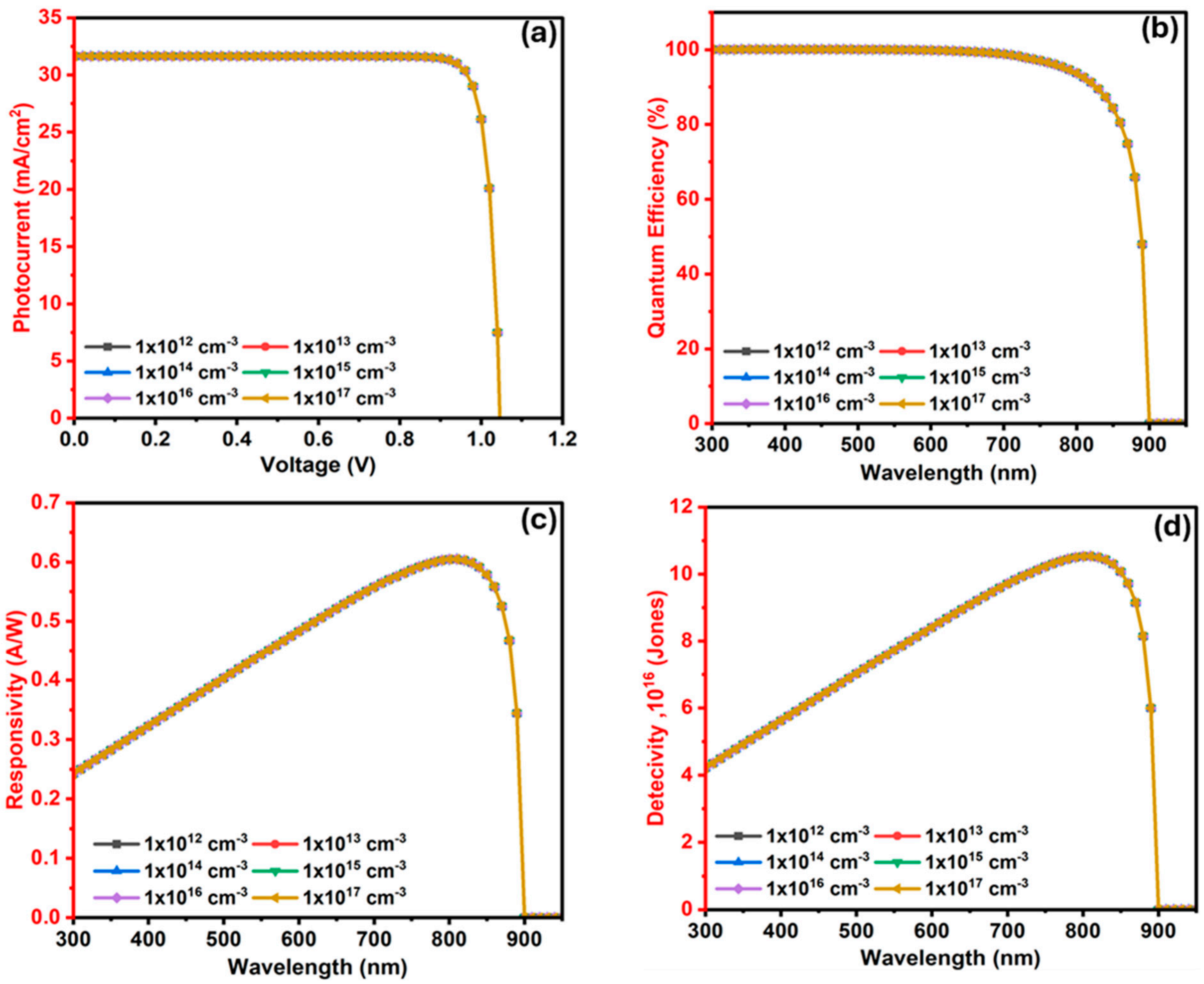

Figure S3. The effect of flaw level (N_t) in the Sb_2S_3-based in level ranging from 1×10^{12} -1×10^{17} cm^{-2} on device performance (a) I–V characteristics (b) QE (c) responsivity (d) detectivity.

5. Influence of In_2S_3/Ba_3SbI_3 junction interface defect on the Ba_3SbI_3 photodetector

To optimize photodetector performance, we investigate the defect density (N_t) at the In_2S_3/Ba_3SbI_3 interface, systematically varying it from 1×10^8 to 1×10^{15} cm^{-2}. Figure S4 shows the influence of interface defect density on the characteristics of the In_2S_3/Ba_3SbI_3 junction. In Figure S4 (a), it is noted that as the interface defect density increases from 1×10^8 to 1×10^{15} cm^{-2}, the Jsc remains nearly constant at 31.655 mA/cm². This signifies that the carrier generation and extraction under short-circuit conditions are largely unaffected by interface recombination. Conversely, the Voc exhibits a noteworthy sensitivity to defect density. It remains fixed at 1.047 V for defect concentrations up to 1×10^{11} cm^{-2}. However, a further increase in defect density from 1×10^{12} to 1×10^{15} cm^{-2} resulted in a gradual decline in Voc from 1.044 V to 0.844 V. This decrease is ascribed to improved Shockley–Read–Hall recombination at the interface, which diminishes the built-in potential across the junction [17].

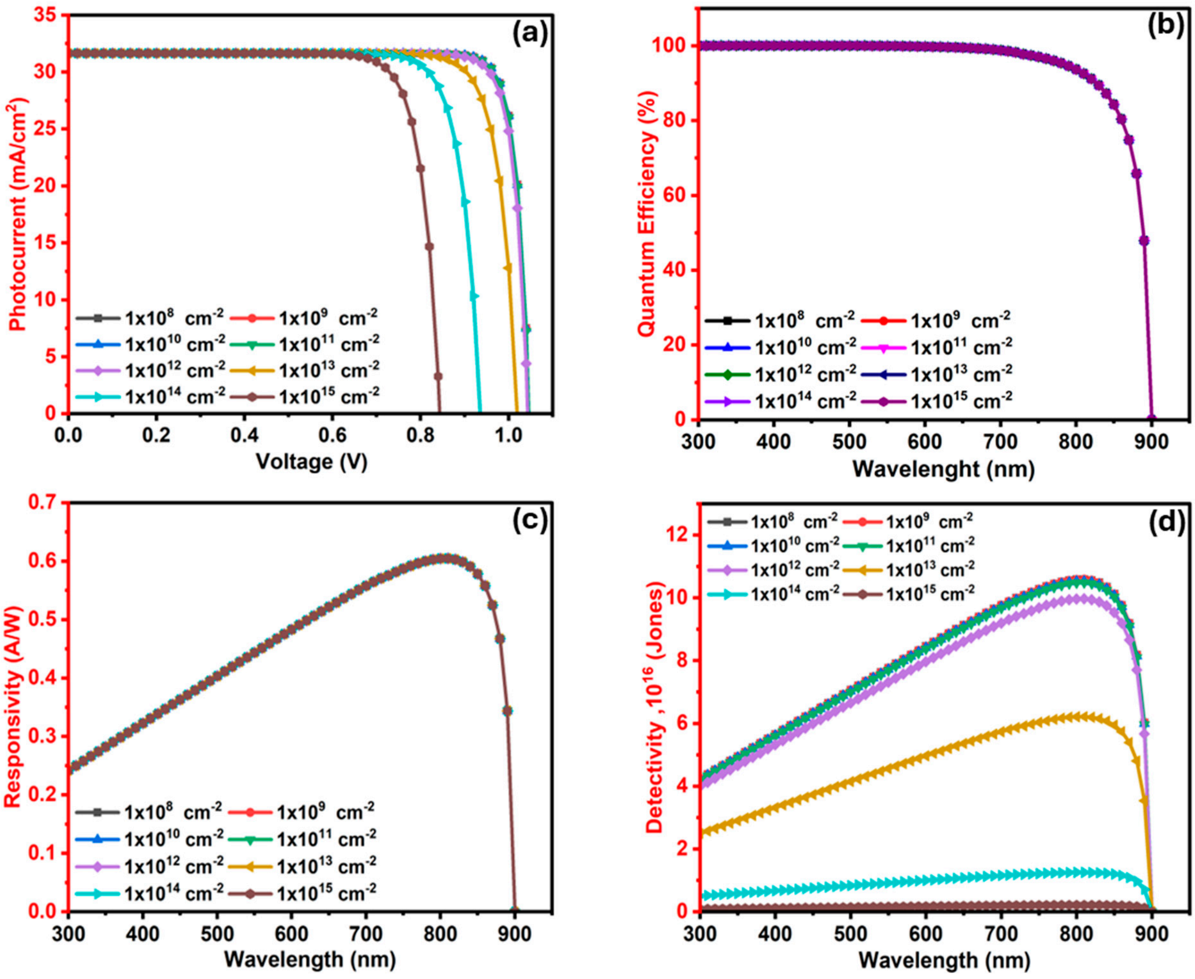

Figure S4. The characteristics of the Ba_3SbI_3 photodetector with respect to the change in the interface flaw at the In_2S_3/Ba_3SbI_3 junction (a) I–V characteristics (b) QE (c) responsivity, and (d) detectivity.

In Figure S4(b), the QE remains stable across the visible range for all defect densities, with a maximum of 100 % in the 300–560 nm range. Beyond 560 nm, QE gradually decreases, reaching ~ 94 % at 800 nm and ~ 80 % at 860 nm, and finally dropping to zero near 900 nm, which corresponds to the band edge of Ba_3SbI_3. Figure S4(c) presents the responsivity characteristics dependent on the wavelength and interface defects. It exhibits the same spectral profile for all defect densities, rising from 0.24 A/W at 300 nm to a peak of 0.605 A/W near 810 nm, before falling sharply at the band edge of 900 nm. This stability confirms that interface defects have minimal influence on the photocurrent generation under same illumination condition discussed in Figure S4(a).

In contrast, detectivity is significantly impacted by interface quality, as indicated in Figure S4(d). For low- interfacial defect densities less than 1×10^{11} cm^{-2}, D* reaches its peak

magnitude of 1.05×10^{17} Jones at 810 nm, while higher defect concentrations result in a marked reduction, falling to 2×10^{15} Jones for 1×10^{15} cm^{-2} at the same wavelength. This decline in D* might be linked to the increased dark current originating from defect-assisted recombination [18]. These results indicate that while the In_2S_3/Ba_3SbI_3 photodetector maintains stable Jsc, QE, and R across a wide range of interface defect densities, Voc and D* degrade significantly beyond 1×10^{11} cm^{-2}. An optimal performance is observed at around 1×10^{10} cm^{-2}, and this value is therefore selected for the subsequent calculations.

5. Influence of Ba_3SbI_3/Sb_2S_3 junction interface defect on the Ba_3SbI_3 photodetector

Figure S5(a) illustrates the influence of interface-defect density on Jsc and Voc for the Ba_3SbI_3/Sb_2S_3 heterojunction. From 10^8 to 10^{13} cm^{-2}, both parameters remain nearly constant, with Jsc at 31.655 mA cm^{-2} and Voc at 1.047 V, as low trap density minimizes Shockley–Read–Hall recombination and ensures efficient carrier collection [17]. A slight Voc drop from 1.0465 to 1.0426 V when the interface defects are between 10^{12} and 10^{13} cm^{-2} marks the onset of recombination losses, while Jsc is unaffected. Once the interface defects increase to 10^{14}–10^{15} cm^{-2}, both parameters deteriorate more noticeably, with Voc decreasing from 1.0197 to 0.971 V and Jsc declining from 31.617 to 31.300 mA.cm^{-2}. The higher interface defect density introduces mid-gap recombination centres, reduces quasi-Fermi level splitting and built-in potential, and enables trap-assisted tunnelling that weakens the voltage and photocurrent [19].

Figure S5(b) presents the QE spectra dependent wavelength and Ba_3SbI_3/Sb_2S_3 interface defects. At low and moderate defect densities spanning from 10^8–10^{13} cm^{-2}, QE is ~100 % from 300 to 550 nm. The gradual decrease beyond 550 nm reflects the reduced absorption coefficient as the photon energy approaches the bandgap, not interface losses. At 10^{14}–10^{15} cm^{-2}, the QE curve shows a slight uniform downward shift, with ~ 1.7 % loss at 800 nm from ~ 93.7 % to ~ 92.0 %. The abrupt cut-off near 900 nm corresponds to the absorber band edge, where photons lack sufficient energy to generate electron–hole pairs.

Figure S5(c) shows the responsivity spectra, which mirrors the QE trends. For defect densities less than 10^{13} cm^{-2}, R rises with wavelength due to the increasing fraction of photons absorbed closer to the depletion region. It reaches its summit magnitude of about 0.605 A W^{-1} at 810 nm. Unlike the In_2S_3 /Ba_3SbI_3 interface, responsivity is slightly reduced at Ba_3SbI_3/Sb_2S_3 interfaces as interfacial defect level ascends to 10^{15} cm^{-2}. Its peak value drops slightly to around 0.594 A W^{-1} at 810 nm, consistent with the QE reduction.

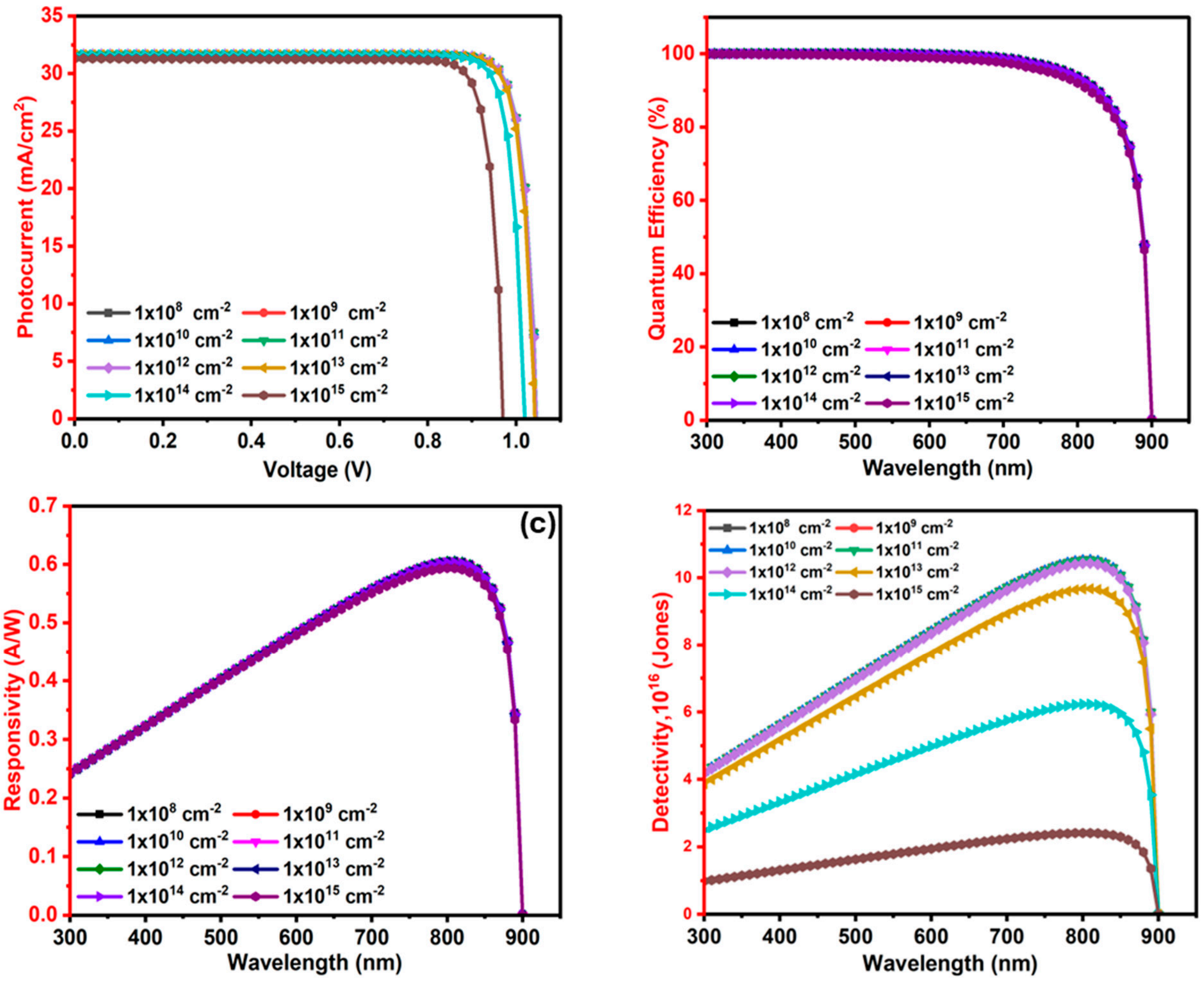

Figure S5. The characteristics of the Ba_3SbI_3 photodetector with the change I the interface defect concentration at the Ba_3SbI_3/Sb_2S_3 interface (a) I–V characteristics (b) QE (c) responsivity, (d) detectivity.

Figure S5(d) illustrates the dependence of detectivity on wavelength and interface defect density in the Ba_3SbI_3/Sb_2S_3 heterojunction photodetector. It appears that detectivity is the parameter most sensitive to interface defects. At 10^8–10^{11} cm^{-2}, D* hikes from 1.03 $\times 10^{17}$ Jones to its peaks of 1.05 $\times 10^{17}$ Jones at 810 nm. As the interface defect density increases, D* declines sharply to 1.04×10^{17} Jones at 10^{12} cm^{-2}, 9.65×10^{16} Jones at 10^{13} cm^{-2}, 6.21×10^{16} Jones at 10^{14} cm^{-2}, and 2.41×10^{16} Jones at 10^{15} cm^{-2}. Such pronounced deterioration is caused by the exponential sensitivity of dark current to recombination-active traps [18].To balance the interface effects across all parameters, the Ba_3SbI_3/Sb_2S_3 interface defect density should be maintained at a moderate level to preserve overall device performance. This condition is achieved when the defect density does not exceed 10^{11} cm^{-2}, while a value of 10^{10} cm^{-2} is considered optimal for subsequent calculations.

6. The total recombination for the structure with and without Sb_2S_3

Recombination in photodetectors occurs when electrons and holes recombine, resulting in the loss of photogenerated charge carriers. In highly optimized photodetectors, the overall device performance is strongly influenced by the carrier density and lifetime, which together govern the recombination rate.

Figure S6 presents the total recombination profile of the Ba_3SbI_9-based photodetector with and without the incorporation of the Sb_2S_3 interfacial layer. The results reveal that introducing Sb_2S_3 significantly suppresses recombination, particularly near the front junction region, where interfacial defects typically dominate carrier loss. The total recombination rate in the Sb_2S_3-modified device decreases by several orders of magnitude compared to the reference sample, clearly indicating the reduction of interface-related recombination pathways. This behaviour confirms that the Sb_2S_3 layer acts as an effective surface passivation material, reducing defect-assisted non-radiative recombination and enhancing charge carrier lifetime. Consequently, carrier transport across the heterojunction is improved, leading to superior photoresponse and enhanced overall device efficiency.

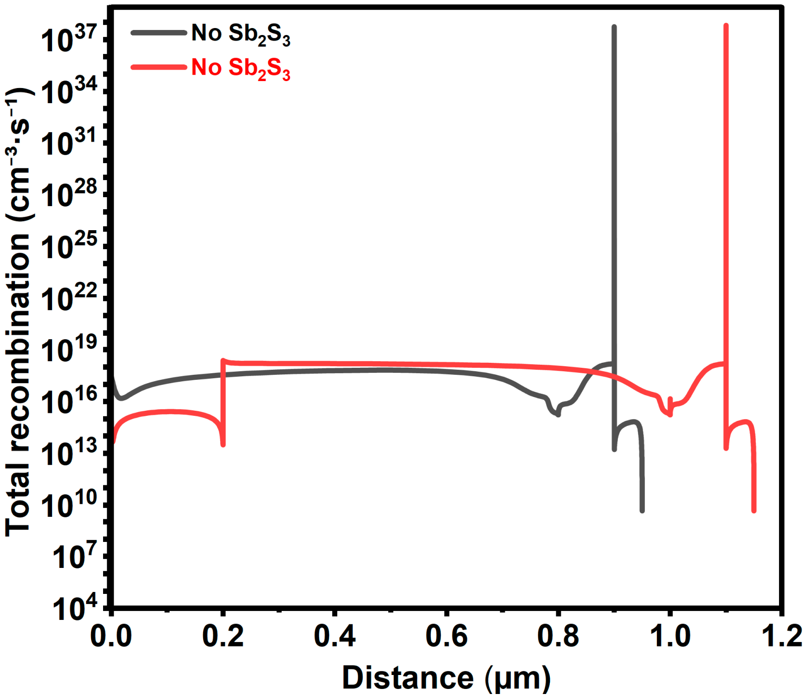

Figure S6. spatial distribution of total recombination rate across the device structure for photodetectors with and without the Sb_2S_3 layer.

7. The series, and shunt resistance impact on the Ba_3SbI_3 photodetector

Figure S6 depicts the influence of series resistance (R_s) and shunt resistance (R_{sh}) on photodetector efficiency. Ideally, the value of the series resistance is zero, while the shunt resistance is infinite. Practically, R_s originates from contact resistance and interfacial barriers between device layers, whereas R_{sh} arises from defect-related recombination and lateral leakage paths [6].This section examines how sweeping the R_s from 0 to 20 Ω.cm² and R_{sh} from 0 to 5 × 10^4 Ω.cm² affects the J–V characteristics of the photodetector.

Figure S7 (a) reveals that R_s has a slight impact on both Jsc and Voc as its value increases. At R_s = 0 Ω.cm², the photodetector displays a Jsc of 1.655 mA/cm², while the Voc reaches 1.044 Volt at the same Rs value. As R_s gradually increases from 0 to 20 Ω.cm², both parameters experience a slight reduction, with Jsc and Voc lessening to 1.655 mA/cm² and 1.044 V, individually. This finding indicates that an elevated R_s introduces ohmic losses, which limit the flow of photogenerated carriers and thereby reduce device sensitivity and efficiency. This behaviour is consistent with reported work on photodetectors [20].

Figure S7 (b) shows the impact of varying R_{sh} from 0 to 5×10^4 Ω.cm² on the photodetector's behaviour. The results indicate that changes in R_{sh} have a negligible effect on Jsc and Voc, which remain constant at 1.047 V and 31.655 mA/cm², respectively. Further increases in R_{sh} do not exert any noticeable improvement. These findings suggest that, to achieve higher performance, R_s should remain at its ideal boundary value of 0 Ω.cm², while R_{sh} must exceed 5 × 10^4 Ω.cm² [21].

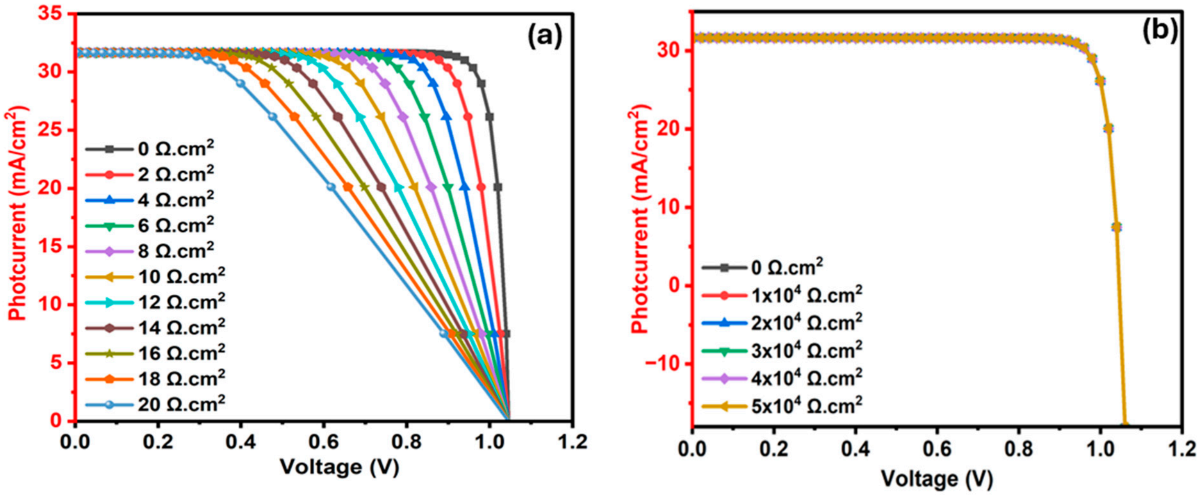

Figure S7. (a) Influence of series resistance on Voc I-V characteristics (b) impact of shunt resistance on I-V curve.

8. Analysis the noise-equivalent power (NEP) of optimized photodetector

The noise-equivalent power (NEP) represents the minimum detectable optical power corresponding to a signal-to-noise ratio of unity. For a shot-noise-limited photodetector, NEP can be expressed as [22]:

$$\mathrm{NEP} = \frac{\sqrt{2qI_d}}{R} \tag{S4}$$

where q is the elementary charge, I_d is the dark current density (mA/cm²), and R is the responsivity (A/W). Using the equation (S4), the calculated NEP is 7.6×10^{-18} W Hz$^{1/2}$.

This remarkably low NEP value confirms the high sensitivity and low-noise characteristics of the proposed photodetector, demonstrating its potential for weak-light detection applications. The obtained NEP is comparable to or even lower than those reported for state-of-the-art photodetectors based on two-dimensional materials and perovskite heterostructures, highlighting the superior performance of the present device [23, 24].

Table S8. Performance comparison of proposed photodetector at different visible and NIR wavelengths.

Parameter	Symbol	Value	Unit
Dark current density	I_d	1.03×10^{-16}	Ma/cm^2
Responsivity	R	0.605	A/W
Elementary charge	q	1.602×10^{-19}	C
Equation		$\mathrm{NEP} = \frac{\sqrt{2qI_d}}{R}$	—

9. The responsivity and the detectivity at different wavelengths in the visible and NIR regions for the optimized photodetector

Table S8 presents the optimized photodetector's photoresponse under visible and near-infrared (NIR) light at various wavelengths, under a constant incident power of 100 mW/cm². The device exhibits the highest responsivity and specific detectivity at 810 nm, outperforming its response at other wavelengths. This enhanced performance is primarily attributed to efficient photon absorption near the absorber's band edge, where the photon energy closely matches the material's bandgap. As a result, carrier generation is improved, leading to a stronger photocurrent response [7].

Table S9. Performance comparison of proposed photodetector at different visible and NIR wavelengths.

Spectral Wavelength (nm)	Responsivity (A/W)	Detectivity (Jones)
400	0.323	6.62×10^{16}
500	0.403	7.03×10^{16}
600	0.483	8.41×10^{16}
700	0.558	9.72×10^{16}
810	0.605	1.05×10^{17}
890	0.344	5.99×10^{16}

10. The proposed synthesis routes for the Ba_3SbI_3 coumpound

The synthesis of Ba_3SbI_3 has not yet been achieved and remains experimentally unexplored. Nevertheless, DFT calculations indicate that this compound is thermodynamically stable and could be realized under appropriate synthesis conditions. Building on these theoretical predictions, we propose a feasible solid-state reaction route for the preparation of Ba_3SbI_3, analogous to those reported for related halide systems. Notably, several ternary halide compounds with similar chemical frameworks have already been synthesized via solid-state reactions between binary precursors of comparable composition [25].For instance, Ca_3AsI_3 has been reported to form when Ca_3As_2 reacts with CaI_2 in a 1:3 molar ratio under annealing at 800 °C [26]. Increasing the reaction temperature to 900 °C, Ca_3AsBr_3 was obtained by combining Ca_3As_2 with $CaBr_2$ in the same stoichiometric proportion [27]. Likewise, Ca_3PCl_3 was produced from Ca_3P_2 and $CaCl_2$ in a 1:3 molar ratio at 1000 °C [28]. Beyond compounds containing heavier elements, this synthetic strategy has also been extended to lighter systems. For example, Mg_3NF_3 was prepared through the reaction between Mg_3N_2 and MgF_2, employing the same 1:3 stoichiometric ratio and a higher annealing temperature of 1050 °C [29]. These examples collectively demonstrate feasibility of similar synthesis pattern among A_3BX_3-type halide materials, in which a binary compound (A_3B_2 or A_3N_2) reacts with the corresponding halide (AX_2) in a 1:3 molar ratio to yield the desired ternary phase.

A feasible route involves reacting Ba_3Sb_2 with BaI_2 in a 1:3 molar ratio under an inert argon atmosphere or within vacuum-sealed quartz ampoules to prevent oxidation and moisture degradation. The mixture can be homogenized thoroughly, pelletized, and annealed at 750–900 °C for 2–24 hours, followed by slow cooling to promote crystallinity. To synthesize Ba_3SbI_3 via a solid-state reaction, the most suitable precursors are Ba_3Sb_2 and BaI_2 in a (1:3) molar

ratio, following the general scheme observed for analogous compounds. The overall reaction can be represented as:

$$Ba_3Sb_2 + 3\,BaI_2 \rightarrow 2\,Ba_3SbI_3 \quad (S5)$$

All procedures involving barium, antimony, and iodine compounds should follow appropriate safety measures. Barium compounds are toxic and must be handled in a fume hood with proper PPE (gloves, lab coat, and goggles). Antimony may cause irritation, especially as fine powders, so dust exposure should be minimized. Iodine compounds such as BaI_2 are hygroscopic and corrosive, requiring storage and handling under dry, inert conditions (e.g., argon glovebox).

11. Supplementary References

[1] A. Ghosh *et al.*, "Innovative double absorber solar cell design combining Ca3AsI3 and Ca3PI3 perovskites for achieving over 29% efficiency," *Optics & Laser Technology,* vol. 183, p. 112399, 2025.

[2] M. A. H. Pappu, M. I. R. Ebon, and J. Hossain, "Design and simulation of BeSiP2-based high-performance solar cell and photosensor," *Solar Energy,* vol. 279, p. 112837, 2024.

[3] M. M. Islam, M. F. Rahman, M. H. Rahman, M. Z. Bani-Fwaz, R. Pandey, and M. Harun-Or-Rashid, "Unlocking the lead-free new all inorganic cubic halide perovskites of Ba3MI3 (M= P, As, Sb) with efficiency above 29%," *Journal of Materials Science,* vol. 59, no. 48, pp. 22109-22131, 2024.

[4] M. N. H. Riyad, A. Sunny, M. M. Khatun, S. Rahman, and S. R. A. Ahmed, "Performance evaluation of WS2 as buffer and Sb2S3 as hole transport layer in CZTS solar cell by numerical simulation," *Engineering Reports,* vol. 5, no. 5, p. e12600, 2023.

[5] A. Kumar *et al.*, "Boosting the efficiency up to 33% for chalcogenide tin mono-sulfide-based heterojunction solar cell using SCAPS simulation technique," *Renewable Energy,* vol. 226, p. 120462, 2024.

[6] M. C. Islam, B. K. Mondal, M. A. H. Pappu, and J. Hossain, "Numerical evaluation and optimization of high sensitivity Cu2CdSnSe4 photodetector," *Heliyon,* vol. 10, no. 17, 2024.

[7] M. I. R. Ebon, M. A. H. Pappu, S. N. Shiddique, and J. Hossain, "Unveiling the potentiality of a self-powered CGT chalcopyrite-based photodetector: theoretical insights," *Optical Materials Express,* vol. 14, no. 4, pp. 907-921, 2024.

[8] M. A. Nalianya *et al.*, "Numerical study of lead free CsSn0. 5Ge0. 5I3 perovskite solar cell by SCAPS-1D," *Optik,* vol. 248, p. 168060, 2021.

[9] S. R. A. Ahmed, "Investigation on the performance enhancement of heterojunction SnS thin-film solar cell with a Zn3P2 hole transport layer and a TiO2 electron transport layer," *Energy & Fuels,* vol. 38, no. 2, pp. 1462-1476, 2023.

[10] F. Horani and E. Lifshitz, "Deciphering the Structural Evolution and Growth Mechanism of 3D β-In2S3 Nanostructures," *The Journal of Physical Chemistry C,* vol. 123, no. 50, pp. 30723-30731, 2019.

[11] X. Ma, Y. Wu, Y. Lv, and Y. Zhu, "Correlation effects on lattice relaxation and electronic structure of ZnO within the GGA+ U formalism," *The Journal of Physical Chemistry C,* vol. 117, no. 49, pp. 26029-26039, 2013.

[12] A. Radzwan, R. Ahmed, A. Shaari, A. Lawal, and Y. X. Ng, "First-principles calculations of antimony sulphide Sb2S3," *Malays. J. Fundam. Appl. Sci.,* vol. 13, no. 3, pp. 285-289, 2017.

[13] M. H. Jameel *et al.*, "A comparative DFT study of bandgap engineering and tuning of structural, electronic, and optical properties of 2D WS2, PtS2, and MoS2 between WSe2, PtSe2, and MoSe2 materials for photocatalytic and solar cell applications," *Journal of Inorganic and Organometallic Polymers and Materials,* vol. 34, no. 1, pp. 322-335, 2024.

[14] S. Abdo *et al.*, "Unveiling the Potential of Novel Ternary Chalcogenide SrHfSe3 for Eco-Friendly, Self-Powered, Near-Infrared Photodetectors: A SCAPS-1D Simulation Study," *Sci,* vol. 7, no. 3, p. 113, 2025.

[15] S. N. Shiddique, A. T. Abir, S. S. Nushin, B. K. Mondal, and J. Hossain, "Numerical probing into the role of experimentally developed ZnTe window layer in high-performance Ag3AuSe2 photodetector," *Results in Materials,* vol. 25, p. 100651, 2025.

[16] M. R. Miah, M. I. R. Ebon, A. T. Abir, and J. Hossain, "Discovering the inherent properties of CdS/TiTe2/Cu2Te near infrared photodetector: A computational analysis," *Next Research,* vol. 2, no. 2, p. 100262, 2025.

[17] P. Jadeja, S. Yadav, A. Ravalia, and S. Katba, "Investigating the impact of various Electron Transport Layers on the performance of Sn-based Perovskite Solar Cells: A Device Simulation using SCAPS-1D," *Journal of Physics and Chemistry of Solids,* p. 113088, 2025.

[18] M. S. Mollah, M. A. H. Pappu, and J. Hossain, "Theoretical design and insight of Fe2GeS4-based optoelectronic devices," *Next Research,* p. 100575, 2025.

[19] M. N. Hasan, M. I. R. Ebon, and J. Hossain, "Numerical Simulation to Achieve High Efficiency in CuTlSe2–Based Photosensor and Solar Cell," *International Journal of Energy Research,* vol. 2025, no. 1, p. 4967875, 2025.

[20] M. I. R. Ebon, A. T. Abir, D. Pathak, and J. Hossain, "Theoretical insights toward a highly responsive AgInSe2 photodetector," *Applied Research,* vol. 3, no. 6, p. e202400038, 2024.

[21] S. Singh, S. Kumar, M. Deo, and R. Chauhan, "Unveiling the potential of organometal halide perovskite materials in enhancing photodetector performance," *Optical and Quantum Electronics,* vol. 55, no. 10, p. 846, 2023.

[22] S. Masanta, C. Nayak, P. Agarwal, K. Das, and A. Singha, "Monolayer Graphene–MoSSe van der Waals Heterostructure for Highly Responsive Gate-Tunable Near-Infrared-Sensitive Broadband Fast Photodetector," *ACS Applied Materials & Interfaces,* vol. 15, no. 11, pp. 14523-14531, 2023.

[23] J. Wang *et al.*, "Self-driven perovskite narrowband photodetectors with tunable spectral responses," *Advanced Materials,* vol. 33, no. 3, p. 2005557, 2021.

[24] H. Arora *et al.*, "Demonstration of a broadband photodetector based on a two-dimensional metal–organic framework," *Advanced Materials,* vol. 32, no. 9, p. 1907063, 2020.

[25] H.-J. Feng and Q. Zhang, "Predicting efficiencies> 25% A3MX3 photovoltaic materials and Cu ion implantation modification," *Applied Physics Letters,* vol. 118, no. 11, 2021.

[26] C. Hadenfeldt and W. Fester, "Reindarstellung und thermische Stabilität von Calcium-phosphid-und-arsenidiodiden: Ca3PI3, Ca3AsI3, Ca2PI und Ca2AsI," *Zeitschrift für anorganische und allgemeine Chemie,* vol. 490, no. 1, pp. 25-30, 1982.

[27] C. Hadenfeldt and P. Schulz, "Darstellung, Struktur und Temperaturabhängigkeit der Phasenbreite der Phase Ca2− xAs1− xBr1+ x und thermisches Verhalten der Verbindung Ca3AsBr3," *Zeitschrift für anorganische und allgemeine Chemie,* vol. 518, no. 11, pp. 77-86, 1984.

[28] C. Hadenfeldt and H. Herdejürgen, "Darstellung, kristallstruktur und thermisches verhalten der calciumphosphidchloride Ca2− xP1− xCl1+ x (0⩽ x⩽ 0, 18) und Ca3PCl3," *Journal of the Less Common Metals,* vol. 124, no. 1-2, pp. 93-103, 1986.

[29] M. A. Brogan, R. W. Hughes, R. I. Smith, and D. H. Gregory, "Structural studies of magnesium nitride fluorides by powder neutron diffraction," *Journal of Solid State Chemistry,* vol. 185, pp. 213-218, 2012.
